# Pentoxifylline, dexamethasone and azithromycin demonstrate distinct age-dependent and synergistic inhibition of TLR- and inflammasome-mediated cytokine production in human newborn and adult blood *in vitro*

**DOI:** 10.1371/journal.pone.0196352

**Published:** 2018-05-01

**Authors:** Esther M. Speer, David J. Dowling, Jianjin Xu, Lukasz S. Ozog, Jaime A. Mathew, Avinash Chander, Donglei Yin, Ofer Levy

**Affiliations:** 1 Department of Pediatrics, Division of Neonatology, Stony Brook University School of Medicine, Stony Brook, New York, United States of America; 2 Department of Medicine, Division of Infectious Diseases, Boston Children’s Hospital, Boston, Massachusetts, United States of America; 3 Harvard Medical School, Boston, Massachusetts, United States of America; 4 Department of Applied Mathematics and Statistics, Stony Brook University, Stony Brook, New York, United States of America; 5 *Precision Vaccine Program*, Boston Children’s Hospital, Boston, Massachusetts, United States of America; University of British Columbia, CANADA

## Abstract

**Introduction:**

Neonatal inflammation, mediated in part through Toll-like receptor (TLR) and inflammasome signaling, contributes to adverse outcomes including organ injury. Pentoxifylline (PTX), a phosphodiesterase inhibitor which potently suppresses cytokine production in newborn cord blood, is a candidate neonatal anti-inflammatory agent. We hypothesized that combinations of PTX with other anti-inflammatory agents, the steroid dexamethasone (DEX) or the macrolide azithromycin (AZI), may exert broader, more profound and/or synergistic anti-inflammatory activity towards neonatal TLR- and inflammasome-mediated cytokine production.

**Methods:**

Whole newborn and adult blood was treated with PTX (50–200 μM), DEX (10^−10^–10^−7^ M), or AZI (2.5–20 μM), alone or combined, and cultured with lipopolysaccharide (LPS) (TLR4 agonist), R848 (TLR7/8 agonist) or LPS/adenosine triphosphate (ATP) (inflammasome induction). Supernatant and intracellular cytokines, signaling molecules and mRNA were measured by multiplex assay, flow cytometry and real-time PCR. Drug interactions were assessed based on Loewe's additivity.

**Results:**

PTX, DEX and AZI inhibited TLR- and/or inflammasome-mediated cytokine production in newborn and adult blood, whether added before, simultaneously or after TLR stimulation. PTX preferentially inhibited pro-inflammatory cytokines especially TNF. DEX inhibited IL-10 in newborn, and TNF, IL-1β, IL-6 and interferon-α in newborn and adult blood. AZI inhibited R848-induced TNF, IL-1β, IL-6 and IL-10, and LPS-induced IL-1β and IL-10. (PTX+DEX) synergistically decreased LPS- and LPS/ATP-induced TNF, IL-1β, and IL-6, and R848-induced IL-1β and interferon-α, while (PTX+AZI) synergistically decreased induction of TNF, IL-1β, and IL-6. Synergistic inhibition of TNF production by (PTX+DEX) was especially pronounced in newborn vs. adult blood and was accompanied by reduction of *TNF* mRNA and enhancement of *IL10* mRNA.

**Conclusions:**

Age, agent, and specific drug-drug combinations exert distinct anti-inflammatory effects towards TLR- and/or inflammasome-mediated cytokine production in human newborn blood *in vitro*. Synergistic combinations of PTX, DEX and AZI may offer benefit for prevention and/or treatment of neonatal inflammatory conditions while potentially limiting drug exposure and toxicity.

## Introduction

Inflammation, driven in part via engagement of pattern recognition receptors (PRRs) by endogenous and exogenous agonists, contributes to multiple perinatal diseases including sepsis, bronchopulmonary dysplasia (BPD) and brain injury [1–3]. Corticosteroids are currently the main anti-inflammatory treatment in neonates but have significant short- and long-term adverse effects such as gastrointestinal and cardiovascular complications, insulin resistance as well as potential growth restriction and neurodevelopmental disability, especially with early use of high-dose dexamethasone (DEX) during the first week of life [4–5]. There is thus an unmet medical need for effective anti-inflammatory alternatives to currently employed corticosteroid-based treatments, which may have less adverse effects in the treatment of neonatal inflammation and prevent its subsequent sequelae.

The xanthine derivative pentoxifylline (PTX) inhibits a range of phosphodiesterases and increases intracellular cyclic adenosine monophosphate (cAMP) in a variety of cells and tissues, thereby suppressing production of pro-inflammatory mediators including tumor necrosis factor (TNF) [6]. Due to its anti-TNF activity, PTX has been studied in animals and human adults and newborns to ameliorate inflammatory conditions such as BPD, meconium aspiration, necrotizing enterocolitis, and hypoxic ischemic encephalopathy [7–11]. PTX is being evaluated as a potential anti-inflammatory adjunct to antibiotics in neonatal sepsis, and several small studies of PTX for this indication raise the possibility that it may decrease all-cause mortality [12–13]. Azithromycin (AZI), a macrolide antibiotic, exerts anti-inflammatory actions through a range of immunomodulatory mechanisms that involve inhibition of pro-inflammatory pathways as well as phenotypic and functional changes of innate immune cells [14–16]. Combination pharmacotherapy is an established approach to maximize desired effects while limiting unwanted adverse effects through dose reductions of the individual compounds [17–18].

Given the need to reduce inflammatory injury in the newborn, we studied age-dependent effects of anti-inflammatory drug combinations in newborn and adult blood *in vitro*. We hypothesized that PTX + DEX or AZI (DEX/AZI) may provide broader, more profound and/or synergistic blunting of neonatal Toll-like receptor (TLR)- and/or inflammasome-mediated inflammatory cytokine production. As a model for infection-induced neonatal inflammation, we stimulated blood with TLR agonists (TLRAs) that activate TLR4 (the endotoxin lipopolysaccharide (LPS)), TLR7/8 (R848, an imidazoquinoline that induces adult-level inflammatory cytokine production in newborn monocytes) [19], or both TLR pathways and the inflammasome including R848 [19] and the combination of LPS and adenosine triphosphate (ATP) [20]. Using these whole blood assays, which preserve the age-specific immunomodulatory soluble and cellular factors naturally present in the newborn [21–23], we compared the anti-inflammatory effects of (PTX+DEX/AZI). We measured stimuli-mediated cytokine mRNA expression, intracellular cytokines, supernatant cytokine concentrations, and monocyte signaling molecules in newborn and adult blood, and calculated estimates for synergistic anti-inflammatory drug interactions. We found that the agents studied exerted distinct inhibitory effects on the different PRR pathways studied that varied with the agent, agent combination and age of study participant. Whereas (PTX+AZI) synergistically inhibited pro-inflammatory cytokine production in both newborn and adult blood, (PTX+DEX) demonstrated more profound synergistic inhibition of LPS- and R848-induced TNF production in newborn vs. adult blood. Our study suggests that the substantial drug (including steroid)-sparing effect of these potent anti-inflammatory agents, especially when used in combination, might offer new approaches for therapeutic benefit in prevention and/or treatment of neonatal inflammatory conditions including sepsis.

## Materials and methods

### Subjects and human blood collection

Placental cord blood was collected from healthy term newborns between 38 to 41 weeks gestation who delivered by Cesarean section without labor and without current infection, including documented intrauterine infection (i.e. absence of clinical chorioamnionitis, prolonged fetal membrane rupture over 12h, clinical or laboratory signs of early-onset sepsis, or culture-proven sepsis of the mother or the newborn) or HIV [20]. Peripheral venous blood was donated with consent from healthy adult donors between 18 to 55 years of age. All blood collections were in accordance to protocols approved by the Institutional Review Board of Stony Brook University, Stony Brook, NY. Written informed consent was obtained from study participants. Cord blood was collected immediately after delivery of the placenta into sterile sodium heparin tubes containing 15 units/ml heparin (Becton Dickinson; Franklin Lakes, NJ) through puncture of the veins on the fetal side of the placenta using sterile techniques, as previously described [20].

### *In vitro* stimulation and treatment of blood samples

Blood samples were kept at room temperature and processed within 2 hours of collection. Blood was diluted 1:1 with sterile pre-warmed (37°C) RPMI 1640 (Life Technologies; Grand Island, NY) and a final volume of 200 μl was added to each well of 96-well tissue culture-treated round-bottom polystyrene plates (Becton Dickinson). Samples were treated with anti-inflammatory agents or their respective vehicle control, stimulated with TLRAs, and cultured for the respective duration under each experiment at 37°C in a humidified incubator at 5% CO_2_. To maximize the relevance of our *in vitro* study, immune-modulators were tested either alone or in combination and within clinically relevant concentration ranges [24–27], including PTX (Tocris; Minneapolis, MN), DEX and AZI (Sigma Aldrich; St. Louis, MO). Ultrapure preparation of LPS from *E*. *coli* O111:B4 (InvivoGen; San Diego, CA) was used for TLR4 stimulation, R848 (InvivoGen) for TLR7/8 stimulation, or LPS followed by 5 mM ATP (Sigma Aldrich) for 20 min for inflammasome induction. All immune-modulators and TLRAs except LPS were verified endotoxin-free (< 0.1 endotoxin units/ml) by the *Limulus amebocyte* lysate assay (Charles River; Wilmington, MA). Upon completion, samples were centrifuged *in situ* at 500 g for 10 min at room temperature. ~120 μl of supernatants per well were carefully collected without disturbing the cell pellets and stored at -80°C for subsequent analysis, as previously described [20]. Duplicate technical replicates were used for all culture experiments and immunoassays, whereas real-time PCR experiments were conducted in triplicate. The number of independently conducted experiments utilizing blood samples from different donors was as indicated for each experimental design. The optimal duration of TLRA stimulation for the different experimental procedures was determined through kinetic studies [20].

### Measurement of cytokine concentrations in culture supernatants

Supernatant cytokine concentrations were determined with Bio-Plex Pro magnetic multiplex assays (Bio-Rad; Hercules, CA) and analyzed on the Bio-Plex 200 system with Bio-Plex Manager 5.0 software (Bio-Rad), which uses the Brendan five-parameter logistic regression for standard curve fitting. Interferon (IFN)-α concentrations were measured by enzyme-linked immunosorbent assay with the VeriKine Human IFN-α Multi-Subtype Serum ELISA kit (PBL Assay Science; Piscataway, NJ). Cytokine concentrations were expressed as a percentage compared to TLRAs alone, which were defined as 100%.

### Real-time PCR

Total RNA was isolated from cultured whole blood after erythrocyte lysis using QIAamp RNA Blood Mini kits (Qiagen; Valencia, CA). Genomic DNA was removed with RNase-free DNase (Qiagen). In order to obtain adequate RNA yields for our experiments, we pooled a total of 5 wells containing 100 μl whole blood mixed with 100 μl RPMI per well for each PCR sample. The concentration and purity of RNA samples was measured with a NanoDrop ND-1000 spectrophotometer (NanoDrop Technologies; Wilmington, DE). The average 260/280 ratio was 2.0 for cord blood and adult blood samples, with most samples falling within the recommended range of 1.9 to 2.1. Few samples with a 260/280 ratio < 1.8 were further purified as per manufacturer instructions. The transcribed cDNA samples were then used for real-time PCR-based measurements of mRNA expression, which were performed in triplicates, loading 5 ng cDNA per 10 μl reaction well. Reverse transcription employed High Capacity cDNA Reverse Transcription kits (Life Technologies; Foster City, CA). Real-time PCR was performed using TaqMan gene expression assays (Life Technologies). Beta actin served as normalization control and was multiplexed into all reaction wells. In order to assure non-interference of the housekeeping gene with the target genes, all gene expression assays were first pretested by comparing their amplification efficiencies as single-plex assay with their amplification efficiencies when multiplexed with the housekeeping gene. Reactions were run on a StepOne Plus Real-time PCR system (Life Technologies) using TaqMan Fast Advanced Master Mix (Life Technologies). Data were analyzed using the delta-delta threshold cycle (ΔΔC_T_) method [28].

### Flow cytometry

Whole blood samples were prepared with the Cytofix/Cytoperm Fixation/Permeabilization kit (BD Biosciences) with (TNF, interleukin (IL)-6, and IL-10) or without (IL-1β, TLR surface expression) addition of Brefeldin A. After pretreatment with anti-inflammatory agents or vehicle control for 2 hours, samples were stimulated with TLRAs and cultured at 37°C in 5% CO_2_ for 6 hours (intracellular cytokines) or 18 hours (TLR expression). Following surface staining, red blood cell lysis employed FACS Lysing Solution (BD). After fixation and permeabilization, samples were stained with monoclonal antibodies (PE-Cy7-conjugated mouse anti-TNF/clone MAb11, PE-conjugated mouse anti-IL-6/clone AS12, APC-conjugated rat anti-IL-10/clone JES3-19F1, PE-conjugated mouse anti-IL-1β/clone AS10, PE-conjugated mouse anti-CD284/clone TF901, all from BD; PE-conjugated mouse anti-TLR7/clone 4G6 (Invitrogen; Carlsbad, CA), FITC-conjugated mouse anti-CD288/clone 44C143 (Invitrogen)) or their corresponding isotype controls. Samples were analyzed on an LSR Fortessa flow cytometer (BD). Compensation beads (BD Comp Beads, BD) were used as single-stain positive and negative controls. Monocytes were gated with forward and side scatter as CD45^+^CD14^+^ cells (PerCP-Cy5.5-conjugated mouse anti-CD45/clone 2D1, FITC-conjugated mouse anti-CD14/clone M5E2 or APC-conjugated mouse anti-CD14/clone MφP9 (BD), respectively). Data from at least 10,000 monocytes were acquired for each condition and analyzed with *Kaluza* version 1.3 software (Beckman Coulter; Jersey City, NJ). The geometric mean fluorescence intensity (MFI) of all monocytes was determined after subtraction of isotype controls.

For measurement of p38 mitogen activated protein kinase (MAPK) and c-Jun N-terminal kinase (JNK) phosphorylation as well as total inhibitor of ĸappa B (IĸB)α, blood samples were pretreated with anti-inflammatory agents or vehicle control for 2 hours and stimulated with TLRAs for 15 min, followed by immediate red blood cell lysis and fixation using Lyse/Fix Buffer (BD). After permeabilization with Perm Buffer II (BD), samples were simultaneously stained with the appropriate surface and intracellular antibodies (AF647-conjugated mouse anti-p38 [pT180/pY182]/clone 36, PE-conjugated mouse anti-JNK [pT183/pY185]/clone N9-66, AF647-conjugated mouse anti-IĸBα/clone 25/IĸBα/MAD-3).

### Caspase-1 activation

Whole blood samples were pretreated with anti-inflammatory agents or vehicle control for 2 hours. Samples were then stimulated with TLRAs and cultured for 1 hour at 37°C in 5% CO_2_ in the presence of Fluorescent Labelled Inhibitor of Caspases (FLICA) reagent (ImmunoChemistry Technologies; Bloomington, MN), a cell-permeable fluorescent reagent that covalently binds to activated caspase-1, and stained for monocyte surface markers. Upon completion of cultures, samples were immediately subjected to red blood cell lysis and fixation using Lyse/Fix Buffer (BD), washed and analyzed on an LSR Fortessa flow cytometer (BD). MFI data from at least 10,000 monocytes, gated with forward and side scatter as CD45^+^CD14^+^ cells, were acquired for each condition as described above, and analyzed with *Kaluza* version 1.3 software (Beckman Coulter). The geometric MFI of all monocytes was determined after subtraction of background fluorescence (no FLICA reagent).

### Statistical analysis

Supernatant cytokine concentrations of samples treated with anti-inflammatory agents were expressed as a percentage compared to cytokine concentrations induced by TLRAs alone, which were defined as 100%. Linear mixed models, which take into account the possible dependence among measurements from the same sample under different drug concentrations, were employed to analyze drug concentration response data for each cytokine, stimulation condition and anti-inflammatory agent independently. Drug concentrations were treated as categorical variables. The covariance structure among drug concentrations was modeled as compound symmetry, which assumes that all distinct members of a cluster are equally correlated with each other, in order to model the correlation among measurements from the same sample. To ensure residual normality, some response data were log transformed prior to entering the model. The presence of interactions for combined anti-inflammatory treatment effects was determined using the Loewe definition of additivity [29], calculating a hierarchy of interaction indices to best fit the synergistic profile, as described by Harbron [30]. 95% confidence intervals for interaction indices were reported.

Flow cytometric MFI data were presented as fold changes normalized to TLRA stimulation alone. Linear mixed models were employed to analyze each combination of stimulation and target analyte independently. The covariance structure among different treatment conditions within the same subject was modeled as compound symmetry. To ensure residual normality, the response data were log transformed before entering the model. Real-time PCR derived ΔΔC_T_ values were used for all gene expression analyses. Linear mixed models were employed to analyze each combination of stimulation condition and gene independently. The covariance structure among different time points within a subject was modeled as compound symmetry. Since mRNA expression for most genes did not significantly differ between newborns and adults, samples from both cohorts were analyzed combined unless otherwise indicated. For graphic presentation only, relative mRNA expression values of samples treated with anti-inflammatory agents were calculated from ΔΔC_T_ values and normalized to TLRA stimulation alone, which was defined as 100%. SAS (SAS Institute; Cary, NC) statistical software was used for analyses and *GraphPad* Prism Version 6.01 (GraphPad Software; San Diego, CA) for graphing of results. Due to the exploratory nature of this study, no multiple testing adjustment was implemented [31]. All statistical tests were two-sided and findings were considered significant at *p < 0*.*05*.

## Results

### PTX, DEX, and AZI inhibited TLR- and/or inflammasome-mediated cytokine production in human newborn and adult blood in a distinct and stimulus-dependent pattern

PTX, DEX, and AZI were used within clinically relevant concentration ranges in newborn and adult whole blood assays [24–27]. Although PTX, DEX, and AZI (S1 Fig) exerted minor effects on cytokine production in unstimulated newborn and adult blood, the overall quantitative effects were small in comparison to those observed in stimulated blood samples. Of note, low concentration of DEX induced a 3-fold increase in IL-1β production in unstimulated blood, whereas high concentration of DEX suppressed the production of this cytokine. IFN-α concentrations were undetectable in unstimulated newborn and adult blood.

**Fig 1:**
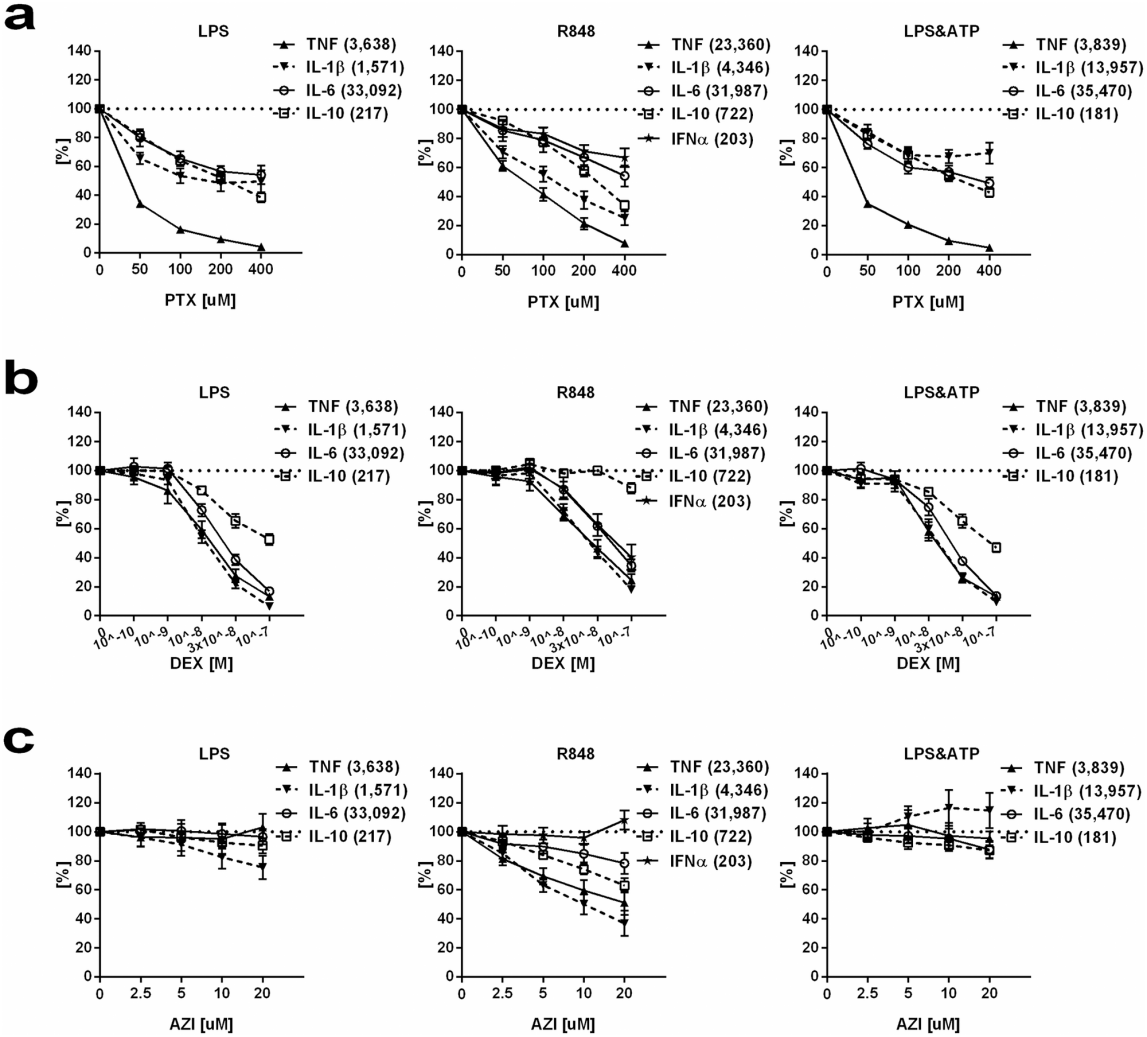
PTX, DEX and AZI inhibit LPS-, R848-, and LPS/ATP-induced cytokine production in newborn cord blood. Human cord blood (n = 10) was pretreated with (a) PTX (50–200 μM), (b) DEX (10^−10^–10^−7^ M), (c) AZI (2.5–20 μM) or vehicle control for 2 hours. Samples were stimulated with 10 ng/ml LPS, 1 μg/ml R848, or LPS followed by 5 mM ATP for inflammasome induction, and cultured for 6 hours at 37°C in 5% CO_2_. Supernatant cytokine concentrations were measured, and results expressed as a percentage (mean ± SEM) compared to TLRAs alone, which were defined as 100%. The corresponding mean cytokine concentrations in pg/ml induced by TLRAs alone were represented in brackets next to each cytokine as part of the graph legends.

As expected, when tested in the absence of anti-inflammatory agents, LPS, R848, and LPS/ATP induced cytokine production in newborn and adult blood (S1 Table). Cytokine concentrations obtained from samples exposed to anti-inflammatory agents were normalized to samples stimulated with TLRAs alone (see S3 Data file for absolute cytokine concentrations). PTX inhibited TLR- and/or inflammasome-mediated pro-inflammatory cytokine production in newborn blood in a concentration-dependent manner, with TNF production inhibited most effectively and potently among the measured cytokines (Fig 1a). DEX inhibited TLR- and/or inflammasome-mediated TNF, IL-1β, IL-6, and IFN-α in newborn blood in a concentration-dependent manner and with comparable efficacy, whereas this agent inhibited IL-10 with diminished efficacy in newborn blood and increased IL-10 in adult blood (Fig 1b, S2 Table). AZI decreased the production of R848-induced cytokines except IFN-α, and at higher concentrations (10 and 20 μM) inhibited LPS-induced production of IL-1β and IL-10 in newborn blood, respectively (Fig 1c and S2 Table). LPS/ATP-induced IL-1β production was increased in newborn blood treated with AZI, while LPS/ATP-induced IL-6 and IL-10 were inhibited at the highest concentration of AZI (20 μM) (Fig 1c and S2 Table).

PTX, DEX, and AZI all modestly decreased the production of the anti-inflammatory cytokine IL-10 in newborn blood, with lower efficacy and potency than the more profound inhibition of pro-inflammatory cytokines TNF in PTX-, TNF, IL-1β, IFN-α, and IL-6 in DEX-, and IL-1β in AZI-treated newborn blood (Fig 1, S2 Fig). Thus the overall effect of these agents was to shift the ratio of cytokines towards a more anti-inflammatory response. In contrast, in adult blood, PTX and DEX increased LPS- and LPS/ATP-induced IL-10 production (S2 Fig and S2 Table). As we have previously reported, PTX inhibited TLR- and/or inflammasome-mediated TNF as well as R848-induced IL-1β and IL-6 with greater efficacy and potency in newborn compared to adult blood [20]. Similarly, PTX inhibited R848-induced IFN-α production with greater efficacy in newborn compared to adult blood (S2 Fig). With the exception of inhibition of TLR- and/or inflammasome-mediated IL-10 production in newborn but not in adult blood treated with DEX (S2 Fig), AZI and DEX did not exhibit any differences in inhibition of cytokine responses between newborn and adult blood samples.

Furthermore, as summarized in Table 1, all agents tested demonstrated stimulus-dependent inhibition of cytokine production. Whereas DEX preferentially inhibited LPS- and LPS/ATP-induced cytokine production in newborn blood, AZI showed significantly greater inhibition of R848-induced cytokines with little effect on LPS/ATP-induced inflammatory cytokine production. PTX, on the other hand, demonstrated enhanced inhibition of LPS- and LPS/ATP-induced compared to R848-induced TNF and IL-6 production, but greater inhibition of R848-induced compared to LPS- and LPS/ATP-induced IL-1β production in newborn cord blood.

**Table 1:** Stimulus-dependent inhibition of cytokine production in newborn blood by PTX, DEX, and AZI.

Cytokine	Anti-inflammatory agent	Comparison of TLR agonist stimulations	Estimated log-difference in slope of cytokine production response curves (95% CI)	p-value ^a^
TNF	PTX	LPS+ATP vs R848	-2.30 (-3.38,-1.21)	<.0001
		LPS vs R848	-2.07 (-3.15,-0.99)	0.0003
	DEX	LPS+ATP vs R848	-7.58 (-10.51,-4.64)	<.0001
		LPS vs R848	-7.07 (-9.92,-4.23)	<.0001
	AZI	LPS+ATP vs R848	0.04 (0.03,0.05)	<.0001
		LPS vs R848	0.04 (0.03,0.05)	<.0001
IL-1β	PTX	LPS+ATP vs R848	3.10 (2.53,3.68)	<.0001
		LPS vs R848	2.07 (1.49,2.64)	<.0001
	DEX	LPS+ATP vs R848	-8.24 (-10.90,-5.59)	<.0001
		LPS vs R848	-12.14 (-14.71,-9.56)	<.0001
	AZI	LPS+ATP vs R848	0.07 (0.06,0.08)	<.0001
		LPS vs R848	0.05 (0.04,0.05)	<.0001
IL-6	PTX	LPS+ATP vs R848	-0.93 (-1.39,-0.47)	0.0001
		LPS vs R848	-0.82 (-1.27,-0.36)	0.0006
	DEX	LPS+ATP vs R848	-9.88 (-11.84,-7.91)	<.0001
		LPS vs R848	-8.38 (-10.29,-6.48)	<.0001
	AZI	LPS+ATP vs R848	0.01 (0.00,0.01)	0.0027
		LPS vs R848	0.01 (0.01,0.02)	<.0001
IL-10	PTX	LPS+ATP vs R848	0.23 (-0.09,0.54)	0.1604
		LPS vs R848	-0.07 (-0.39,0.24)	0.6406
	DEX	LPS+ATP vs R848	-7.06 (-8.24,-5.88)	<.0001
		LPS vs R848	-6.06 (-7.21,-4.92)	<.0001
	AZI	LPS+ATP vs R848	0.02 (0.01,0.02)	<.0001
		LPS vs R848	0.02 (0.02,0.02)	<.0001

Cord blood (n = 10) was pretreated with increasing concentrations of PTX (50–200 μM), DEX (10^−10^–10^−7^ M), AZI (2.5–20 μM) or vehicle control for 2 hours. Samples were stimulated with 10 ng/ml LPS, 1 μg/ml R848, or LPS followed by 5 mM ATP for inflammasome induction, and cultured for 6 hours at 37°C in 5% CO_2_. Supernatant cytokine concentrations were measured, and results were normalized to TLRAs alone, defined as 100%. Table 1 compares the changing rates with increasing drug concentrations (i.e., the estimated log difference in slope) of LPS- or LPS/ATP-induced compared to R848-induced cytokine production response curves for each anti-inflammatory agent. Linear mixed models were developed to analyze each cytokine and anti-inflammatory treatment independently. Drug concentrations were analyzed as continuous variables. To model the correlation among measurements from the same sample, the covariance structure between stimulation conditions within the same subject was modeled as compound symmetry, which assumes that all distinct members of a cluster are equally correlated with each other. To ensure residual normality, data were log transformed before entering the model. Confidence interval (CI).

^a^ p-values were based on linear mixed model t-tests.

### Effect of timing of addition of PTX, DEX, and AZI on inhibition of TLR-mediated cytokine production in newborn and adult blood

**Fig 2:**
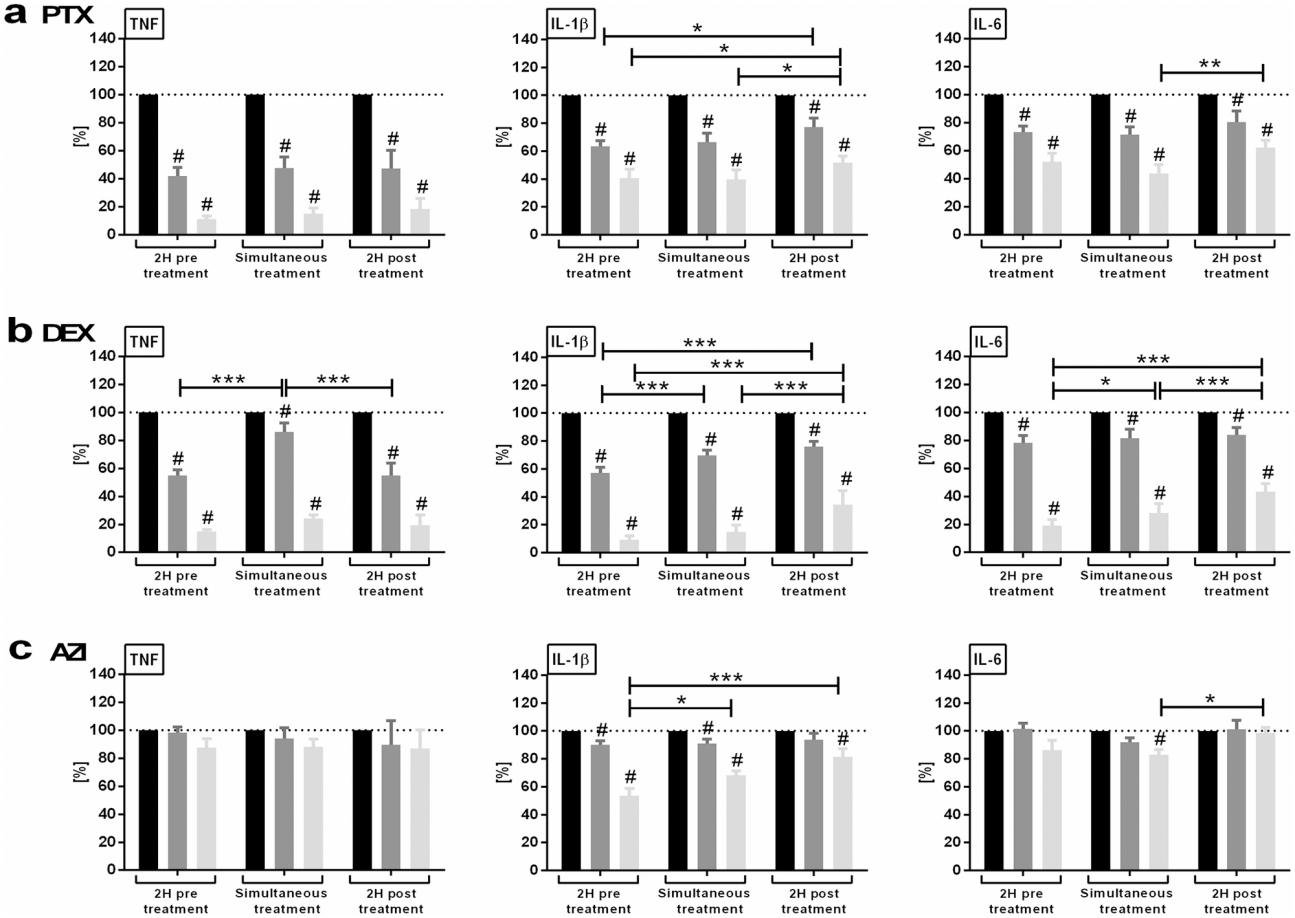
Timing of anti-inflammatory treatment effects extent of inhibition of LPS-induced inflammatory cytokine production. Newborn cord and adult peripheral blood were stimulated with 10 ng/ml LPS. Samples were treated with PTX (50 or 200 μM), DEX (10^−8^ or 10^−7^ M), AZI (2.5 or 20 μM) or vehicle control, which were added either 2 hours before, simultaneously, or 2 hours after TLR stimulation. Following LPS stimulation, samples were cultured for further 6 hours. Supernatant cytokines were expressed as a percentage compared to TLRAs alone, defined as 100%. For delayed treatment samples, cytokine concentrations measured at the start of anti-inflammatory treatment were subtracted from the corresponding delayed treatment samples, in order not to underestimate the delayed treatment effects. Effects of timing and concentration of (a) PTX [Fig 2a adapted from 20, *Speer EM*, *et al*. *Pediatr Res*. *2017;81*: *806–816*], (b) DEX, and (c) AZI on LPS-induced cytokine production (mean ± SEM) in newborn and adult blood combined. To determine significant differences between time points (*p<0.05, **p<0.01, and ***p<0.001), linear mixed models were employed to analyze each cytokine and stimulation independently. The covariance structure between anti-inflammatory concentrations within a subject was modeled as unstructured variance, and the covariance structure among time points within the same concentration was modeled as compound symmetry. Significant changes in cytokine production of samples treated with high (light gray columns) or low (dark gray columns) concentrations of anti-inflammatory agents vs. untreated samples (black columns), calculated from log transformed raw pg/ml values, were indicated as #. Linear mixed models were developed to analyze each cytokine and time point independently as described above, with p<0.05 considered significant. N = 5 per age group.

**Fig 3:**
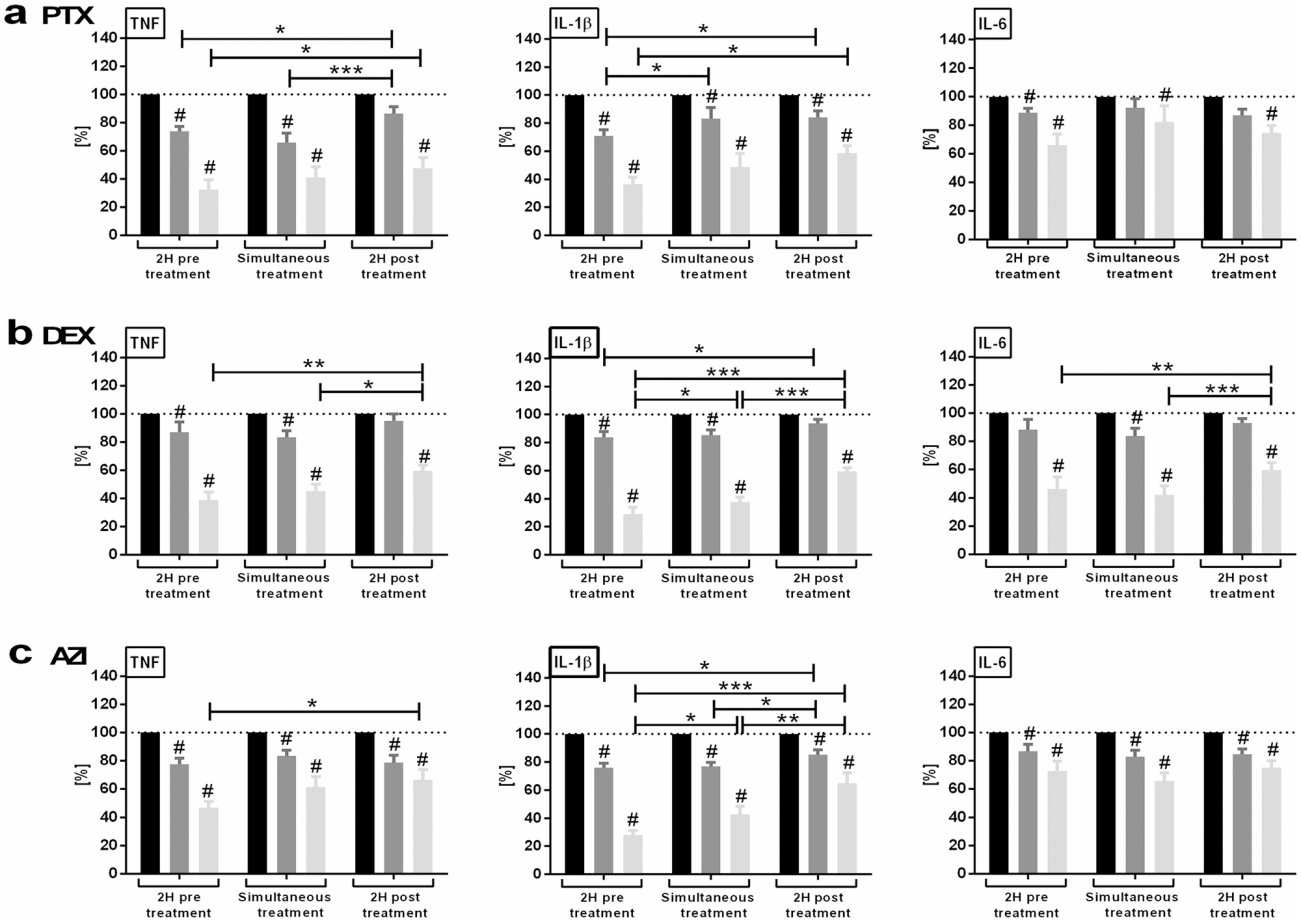
Timing of anti-inflammatory treatment effects extent of inhibition of R848-induced inflammatory cytokine production. Newborn cord and adult peripheral blood were stimulated with 1 μg/ml R848. Samples were treated with PTX (50 or 200 μM), DEX (10^−8^ or 10^−7^ M), AZI (2.5 or 20 μM) or vehicle control, which were added either 2 hours before, simultaneously, or 2 hours after TLR stimulation. Following R848 stimulation, samples were cultured for further 6 hours. Supernatant cytokines were expressed in percent compared to TLRAs alone, defined as 100%. For delayed treatment samples, cytokine concentrations measured at the start of anti-inflammatory treatment were subtracted from the corresponding delayed treatment samples, in order not to underestimate the delayed treatment effects. Effects of timing and concentration of (a) PTX [Fig 3a adapted from 20, *Speer EM*, *et al*. *Pediatr Res*. *2017;81*: *806–816*], (b) DEX, and (c) AZI on R848-induced cytokine production (mean ± SEM) in newborn and adult blood combined. To determine significant differences between time points (*p<0.05, **p<0.01, and ***p<0.001), linear mixed models were employed to analyze each cytokine and stimulation independently. The covariance structure between anti-inflammatory concentrations within a subject was modeled as unstructured variance, and the covariance structure among time points within the same concentration was modeled as compound symmetry. Significant changes in cytokine production of samples treated with high (light gray columns) or low (dark gray columns) concentrations of anti-inflammatory agents versus untreated samples (black columns), calculated from log transformed raw pg/ml values, were indicated as #. Linear mixed models were developed to analyze each cytokine and time point independently as described above, with p<0.05 considered significant. N = 5 per age group.

In order to assess the impact of the timing of administration of anti-inflammatory agents, we further determined if delayed addition of anti-inflammatory agents in relation to TLR stimulation as compared to pretreatment or simultaneous administration would still be effective. PTX (50 or 200 μM) and DEX (10^−8^ or 10^−7^ M) inhibited TNF, IL-1β, and IL-6 production in newborn and adult blood whether added 2 hours before, simultaneously, or 2 hours after LPS (Fig 2) and R848 stimulation (Fig 3), albeit with diminished efficacy and potency with delayed treatment. AZI (2.5 or 20 μM), on the other hand, inhibited LPS- and R848-induced IL-1β and R848-induced TNF and IL-6 production when added 2 hours before, simultaneously, or 2 hours after TLRA stimulation, but did not diminish LPS-induced TNF and IL-6 production (Figs 2 and 3).

### (PTX+DEX/AZI) synergistically inhibited TLR/inflammasome-mediated cytokine production in newborn and adult blood

Synergistic drug combinations may provide comparable treatment effects at lower concentrations of the individual compounds, thus potentially minimizing adverse effects. The determination of drug interactions for combined anti-inflammatory treatment effects for (PTX+DEX) or (PTX+AZI) were assessed based on Loewe’s definition of additivity [29], calculating a hierarchy of interaction indices to best fit the synergistic profile [30]. S3 Table showed “goodness of fit” tests for model fitting compared to the additive model and between hierarchical synergy models for all converged models. Except for (PTX+AZI) effects on R848-induced IFN-α in newborn and adult blood, for which no model converged, all other tested drug combinations and scenarios resulted in at least one converged drug interaction model suggesting that at least one drug synergy model could be fitted in addition to the additive model. Estimates for interaction indices along with 95% CIs were calculated for all converged models (S3 Table). Interaction indices below 1 indicated synergy, with lower values representing higher levels of synergy. Table 2 showed the summary of all statistically significant synergistic anti-inflammatory drug interactions for combined (PTX+DEX) or (PTX+AZI) for the inhibition of LPS-, R848-, and LPS/ATP-induced production of TNF, IL-1β, IL-6, and IFN-α. (PTX+DEX) exerted highly synergistic inhibition of LPS- and LPS/ATP-induced TNF, IL-1β, and IL-6 production and inhibition of R848-induced IL-1β and IFN-α in newborn and adult blood, with parameter estimates (values between 0 and 1 indicate synergy, with lower values indicating greater synergy) ranging between 0.22 (LPS/ATP-induced IL-1β) and 0.55 (LPS-induced TNF production) for the lowest PTX concentration tested (50 μM), when linearly varying responses over concentrations of PTX. Combinations of low concentrations of (PTX+AZI) demonstrated significant synergistic suppression of LPS-induced TNF and IL-1β, LPS/ATP-induced TNF and IL-6, as well as R848-induced IL-1β and IFN-α production in human blood.

**Table 2:** Summary of statistically significant synergistic anti-inflammatory combinations.

Drug combination	Scenario	Model	Synergistic drug concentrations	Interaction index (95% CI) ^a^
PTX & DEX	LPS → TNF	Linearly varying over doses of PTX	50μM PTX	0.55 (0.47–0.63)***
			100μM PTX	0.78 (0.71–0.86)***
PTX & DEX	LPS → IL-1β	Linearly varying over doses of PTX	50μM PTX	0.50 (0.42–0.57)***
			100μM PTX	0.80 (0.71–0.90)***
PTX & DEX	LPS → IL-6	Linearly varying over doses of PTX	50μM PTX	0.39 (0.29–0.48)***
			100μM PTX	0.69 (0.58–0.81)***
PTX & DEX	LPS/ATP → TNF	Linearly varying over doses of PTX	50μM PTX	0.36 (0.29–0.43)***
			100μM PTX	0.66 (0.57–0.76)***
PTX & DEX	LPS/ATP → IL-1β	Linearly varying over doses of PTX	50μM PTX	0.22 (0.17–0.28)***
			100μM PTX	0.56 (0.45–0.68)***
PTX & DEX	LPS/ATP → IL-6	Linearly varying over doses of PTX	50μM PTX	0.37 (0.28–0.46)***
			100μM PTX	0.67 (0.56–0.79)***
PTX & DEX	R848 → IL-1β	Linearly varying over doses of PTX	50μM PTX	0.43 (0.35–0.50)***
			100μM PTX	0.74 (0.65–0.83)***
PTX & DEX	R848 → IFN-α	Linearly varying over doses of PTX	50μM PTX	0.31 (0.22–0.39)***
			100μM PTX	0.66 (0.53–0.79)***
PTX & AZI	LPS → TNF	Separately for each dose combination	50μM PTX & 2.5 μM AZI	0.72 (0.58–0.89)**
PTX & AZI	LPS → IL-1β	Linearly varying over doses of AZI	2.5 μM AZI	0.80 (0.63–0.97)*
PTX & AZI	LPS/ATP → TNF	Separately for each dose combination	50μM PTX & 2.5 μM AZI	0.77 (0.60–0.98)*
PTX & AZI	LPS/ATP → IL-6	Separately for each dose combination	50μM PTX & 2.5 μM AZI	0.40 (0.31–0.51)***
			50μM PTX & 5 μM AZI	0.55 (0.44–0.69)***
			100μM PTX & 2.5 μM AZI	0.57 (0.46–0.71)***
			100μM PTX & 5 μM AZI	0.73 (0.59–0.91)**
PTX & AZI	R848 → IL-1β	Linearly varying over doses of AZI	2.5 μM AZI	0.79 (0.64–0.93)**
PTX & AZI	R848 → IL-6	Linearly varying over doses of AZI	2.5 μM AZI	0.81 (0.63–0.99)*

The determination of drug interactions for combined anti-inflammatory treatment effects were based on Loewe’s definition of additivity [29] by calculating a hierarchy of interaction indices to best fit the synergistic model, as described [30]. A common model across all concentration combinations, a model of linearly varying values over concentration levels of one of the compounds, a model of separately varying values for each concentration level of one of the compounds, and a model of separate values for each concentration combination were fitted for each drug combination and compared to the additive model. To evaluate the performance between models, a set of hierarchical goodness of fit tests were applied. PTX and AZI each had 4 different concentration levels, while DEX had 5 concentrations levels. Each drug combination dataset included cytokine concentration values measured in whole blood culture supernatants from 10 subjects (5 newborns and 5 adults; 8 newborns and 8 adults for LPS- and R848-induced TNF and IL-1β treated with combined PTX and DEX). The drug synergy determinations were based on results from all subjects combined. Estimates for interaction indices along with 95% CIs were calculated for the different drug combinations, treatment scenarios, and synergy models. Interaction indices below 1 indicated synergy, with lower values representing higher levels of synergy. Negative values of drug effects, i.e. cytokine concentrations of treated samples were above those of untreated samples, were removed. The SAS NLIN procedure, which estimates the parameters by nonlinear least squares or weighted nonlinear least squares, was used to fit the regression models [32]. Table 2 provides a summary of the statistically significant synergistic anti-inflammatory combinations. For a complete listing of parameter estimates for all drug combinations, treatment scenarios and synergy models see S3 and S4 Tables.

^a^ p-values were based on t-tests (*p<0.05, **p<0.01, ***p<0.001).

**Fig 4:**
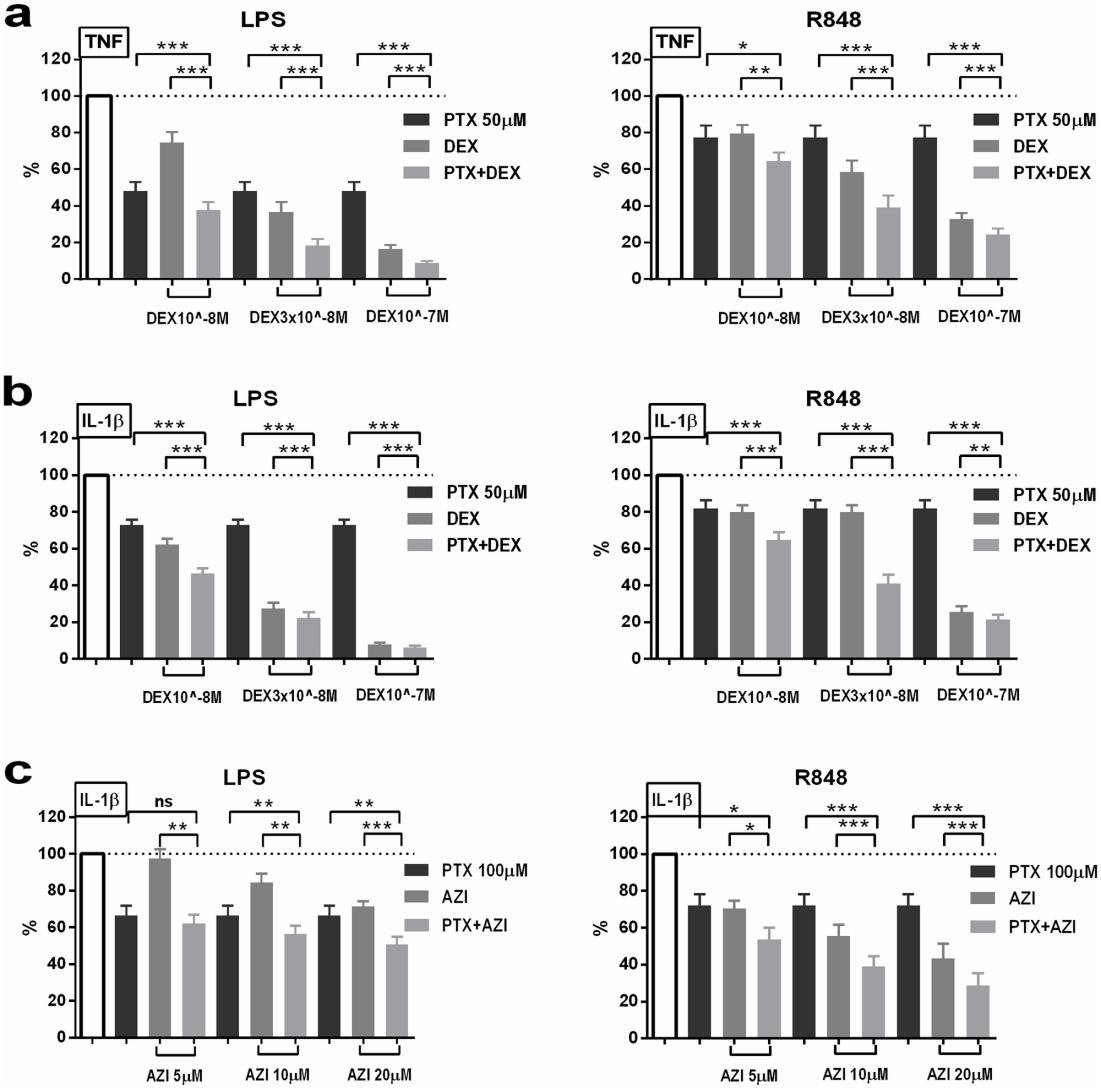
(PTX+DEX) and (PTX+AZI) synergistically inhibit TLR-mediated pro-inflammatory cytokine production in whole blood assays. Newborn cord and adult peripheral blood (n = 8 per cohort for (PTX+DEX) and n = 5 per cohort for (PTX+AZI), analyzed combined) was pretreated with vehicle control, PTX (50–100 μM), DEX (10^−8^–10^−7^ M), AZI (5–20 μM) or combined (PTX+DEX) or (PTX+AZI) for 2 hours. Samples were stimulated with 10 ng/ml LPS or 1 μg/ml R848, and cultured for 6 hours at 37°C in 5% CO_2_. Supernatant cytokine concentrations were measured, and results expressed as a percentage (mean ± SEM) compared to TLRAs alone, which were defined as 100% (see S8 Data file for absolute cytokine concentrations). Synergistic inhibition of LPS- and R848-induced (a) TNF and (b) IL-1β production employing selected concentrations of (PTX+DEX) and (c) IL-1β production with (PTX+AZI). Significant differences based on paired t-tests between single anti-inflammatory agents and their corresponding combinations of agents at the same concentrations as the individual compounds were indicated: *p<0.05, **p<0.01, ***p<0.001.).

As graphically demonstrated in Fig 4, (PTX+DEX) synergistically and potently inhibited LPS- and R848-induced TNF and IL-1β production and (PTX+AZI) synergistically inhibited LPS- and R848-induced IL-1β production at different dose combinations of these agents in whole blood assays. Several combinations of these anti-inflammatory agents at different concentrations reduced pro-inflammatory cytokine concentrations to ~50% of the corresponding lower concentration of the individual compounds.

### Combined (PTX+DEX) demonstrated greater synergistic inhibition of LPS- and R848-induced TNF production in newborn compared to adult blood

As PTX had a significantly stronger inhibitory effect on LPS- and R848-induced TNF and R848-induced IL-1β production in newborn compared to adult blood [20], we next determined if the synergistic effects of combined (PTX+DEX) on inhibition of LPS- and R848-induced TNF and IL-1β production differed between newborn and adult samples. When separately fitting interaction indices of combined anti-inflammatory treatment for each cohort, (PTX+DEX) demonstrated greater synergistic effects on LPS- and R848-induced TNF for 50 and 100 μM PTX in newborn compared to adult blood (Table 3). When linearly varying responses over PTX concentrations, the estimated interaction indices for newborn blood were lower than those for adult blood (e.g. 0.43 [95% CI: 0.35–0.52] versus 0.64 [0.53–0.76] for inhibition of LPS-induced TNF and 0.39 [0.30–0.49] versus 0.49 [0.36–0.61] for suppression of R848-induced TNF production at 50 μM PTX, respectively), indicating greater synergy for this drug combination in newborn samples (Table 3). Goodness of fit-testing comparing the synergy model of separate newborn and adult subject cohorts with the original model of combined newborn and adult data showed significant model improvement (Table 4). The estimated interaction indices for newborn cord blood using this synergy model were significantly lower than those for adult blood (estimated coefficient of covariate d = -0.21, p < 0.001 for LPS-induced and estimated d = -0.18, p < 0.001 for R848-induced TNF inhibition, respectively) (Table 5), indicating greater synergy for newborn samples.

**Table 3:** Higher levels of synergistic inhibition of TLR-mediated TNF and IL-1β production in newborn cord blood with combined (PTX+DEX).

Scenario	Reference drug for (PTX+DEX)	Reference concentration [μM]	Newborns		Adults	
			Interaction index	p-value ^a^	Interaction index	p-value ^a^
LPS → TNF	PTX	**50**	**0.43 (0.35–0.52)**	**<0.0001**	**0.64 (0.53–0.76)**	**<0.0001**
		**100**	**0.70 (0.61–0.80)**	**<0.0001**	**0.81 (0.71–0.92)**	**0.0004**
		200	0.97 (0.83–1.12)	0.7190	0.98 (0.84–1.12)	0.7725
		400	1.24 (1.04–1.45)	0.0194	1.15 (0.96–1.34)	0.1285
R848 → TNF	PTX	**50**	**0.39 (0.30–0.49)**	**<0.0001**	**0.49 (0.36–0.61)**	**<0.0001**
		**100**	**0.71 (0.59–0.83)**	**<0.0001**	**0.74 (0.61–0.86)**	**<0.0001**
		200	1.03 (0.85–1.21)	0.7271	0.98 (0.81–1.15)	0.8493
		400	1.35 (1.11–1.60)	0.0053	1.23 (1.00–1.47)	0.0525
LPS → IL-1β	PTX	**50**	**0.46 (0.35–0.57)**	**<0.0001**	**0.64 (0.52–0.76)**	**<0.0001**
		**100**	**0.80 (0.66–0.94)**	**0.0056**	**0.85 (0.73–0.97)**	**0.0142**
		200	1.15 (0.95–1.35)	0.1495	1.07 (0.91–1.22)	0.4117
		400	1.50 (1.21–1.78)	0.0006	1.28 (1.06–1.50)	0.0126
R848 → IL-1β	PTX	**50**	**0.52 (0.40–0.63)**	**<0.0001**	**0.49 (0.38–0.59)**	**<0.0001**
		**100**	**0.82 (0.69–0.94)**	**0.0050**	**0.76 (0.65–0.87)**	**<0.0001**
		200	1.12 (0.94–1.30)	0.1915	1.03 (0.88–1.19)	0.6725
		400	1.42 (1.17–1.67)	0.0009	1.31 (1.09–1.53)	0.0062

The determination of drug interactions for combined anti-inflammatory treatment effects was based on Loewe’s definition of additivity [29] as described by Harbron [30]. Newborn and adult data were fitted independently for each drug combination and scenario by the following 5 models and compared to the additive (null) model: common model across all concentration combinations, model of linearly varying interaction values over concentrations of one of the compounds, model of separately varying interaction values for each concentration level of one of the compounds, and model of separate interaction values for each concentration combination. The most advanced model which satisfied the following criteria was selected for each scenario as the best model for further analysis: (1) the model could be converged for both newborn and adult data. (2) At least one drug concentration showed synergistic anti-inflammatory effects, i.e. showed an interaction index < 1. The selected model was then modified to examine the difference in synergy between newborns and adults (n = 8 subjects per cohort). The model linearly varying responses over PTX concentrations was the only model satisfying all selection criteria. Goodness of fit-tests comparing the updated models with the original model were then employed. Results for statistically significant synergistic anti-inflammatory drug concentrations are indicated in **bold**.

^a^ p-value was based on t-test.

**Table 4:** Goodness of fit of PTX and DEX synergy model for separate newborn and adult cohorts.

Scenario	Model	DF	Sum of Squares	Test df	Test Sum of Squares	F	p-value ^a^
LPS → TNF	**NULL model** Interaction index = alpha+beta*log(drug concentration)	421	63.42	.	.	.	.
	**Model with cohort** Interaction index = alpha+beta*log(drug concentration)+ d*cohort	420	58.88	1	4.54	32.35	<0.0001
R848 → TNF	**NULL model** Interaction index = alpha+beta*log(drug concentration)	418	82.48	.	.	.	.
	**Model with cohort** Interaction index = alpha+beta*log(drug concentration)+ d*cohort	417	79.33	1	3.14	16.51	<0.0001
LPS → IL-1β	**NULL model** Interaction index = alpha+beta*log(drug concentration)	426	78.31				
	**Model with cohort** Interaction index = alpha+beta*log(drug concentration)+ d*cohort	425	78.22	1	0.09	0.50	0.4815
R848 → IL-1β	**NULL model** Interaction index = alpha+beta*log(drug concentration)	421	75.56				
	**Model with cohort** Interaction index = alpha+beta*log(drug concentration)+ d*cohort	411	69.15	1	6.41	38.10	<0.0001

^a^ p-value was based on F test.

**Table 5:** Parameter estimate for PTX and DEX synergy model for separate newborn and adult cohorts.

Scenario	Synergy model	Parameter	Estimate	Std Err	p-value ^a^
LPS → TNF	**Model with cohort** Interaction index = alpha+beta*log(drug concentration) + d*cohort	d	-0.21	0.04	<0.0001
R848 → TNF		d	-0.18	0.04	<0.0001
LPS → IL-1β		d	-0.03	0.04	0.4853
R848 → IL-1β		d	-0.24	0.04	<0.0001

^a^ p-value was based on t-test.

### PTX, DEX and AZI, alone or in combination, inhibited TLR- and/or inflammasome-mediated pro-inflammatory cytokine mRNA expression in newborn and adult blood and intracellular cytokines in newborn monocytes while preserving or enhancing IL-10

**Fig 5:**
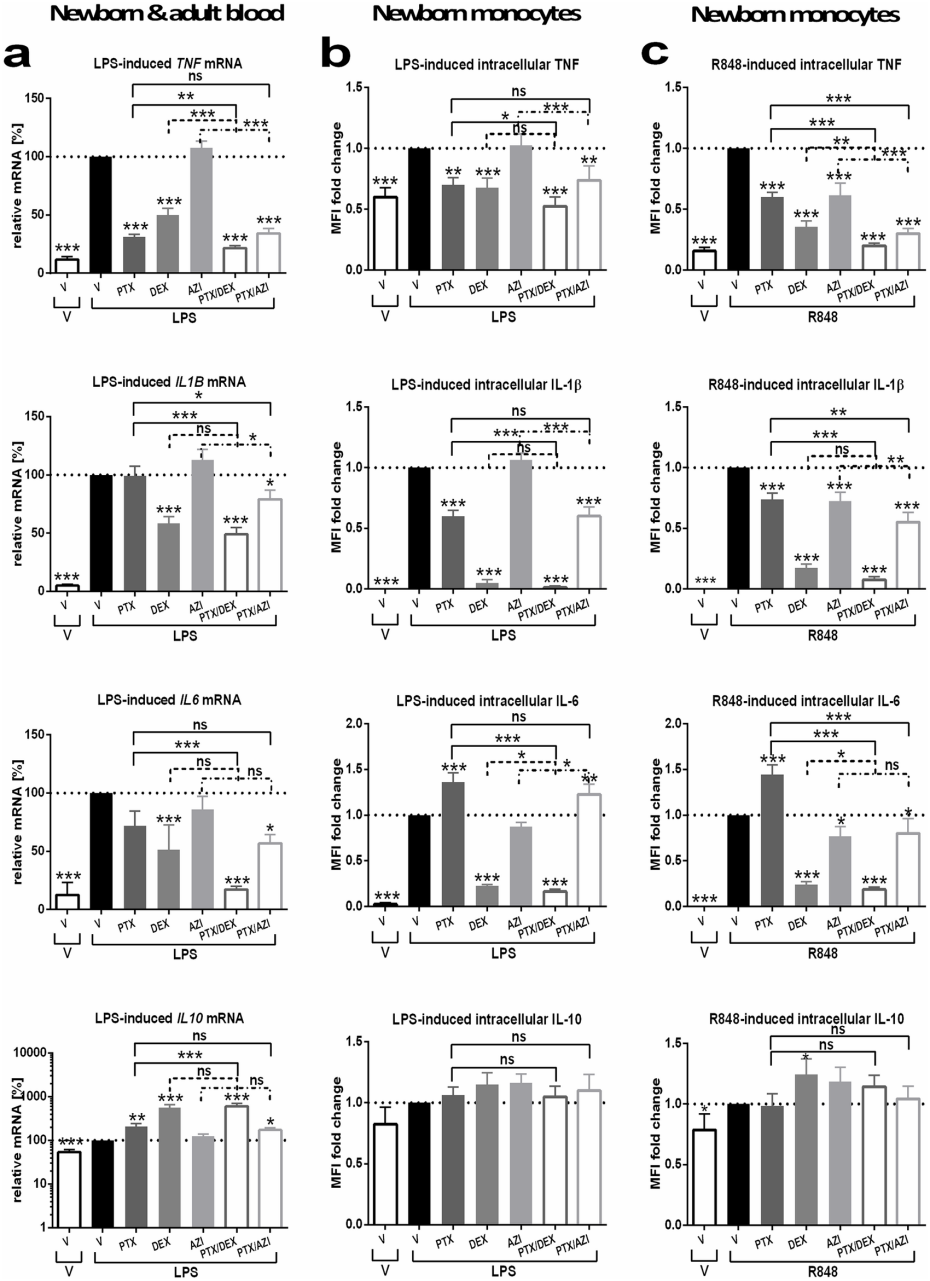
PTX, DEX and AZI demonstrate distinct inhibition of TLR-mediated cytokine mRNA and intracellular protein expression. Whole blood was pretreated for 2 hours with PTX (200 μM), DEX (10^−7^ M), AZI (20 μM) or vehicle control (V), either alone or in combination. Samples were stimulated with 10 ng/ml LPS or 1 μM R848, and cultured for 2 hours (mRNA expression) or 6 hours (flow cytometry) at 37°C in 5% CO_2_, in the presence (TNF, IL-6 and IL-10) or absence (IL-1β) of Brefeldin A for flow cytometric assays. MFI of monocytes, gated with forward and side scatter as CD45^+^CD14^+^ cells, was measured as described in *Methods*. (a) LPS-induced relative mRNA expression (mean ± SEM) in cord and adult blood (n = 5 each, analyzed combined) in response to anti-inflammatory agents compared to LPS-stimulation alone, defined as 100%. Effects of anti-inflammatory treatment on (b) LPS- and (c) R848-induced intracellular cytokines in newborn monocytes (n = 8), plotted as MFI fold changes (± SEM) compared to TLRA stimulation alone (see S6 Data file for raw MFI data). Significant differences based on linear mixed models were indicated: *p<0.05, **p<0.01, ***p<0.001.

PTX and DEX, alone or in combination, decreased TLR- and/or inflammasome-induced *TNF* and increased *IL10* mRNA in newborn and adult blood (Fig 5a, S5 and S6 Tables), whereby (PTX+DEX) exerted significantly greater changes on *TNF* and *IL10* mRNA expression than either agent alone. Similarly, AZI led to greater R848-induced *TNF* mRNA inhibition when used in combination with PTX (S5 and S6 Tables). (DEX±PTX) and (PTX+AZI) decreased TLR- and/or inflammasome-induced *IL1B* and *IL6* mRNA (Fig 5a, S5 and S6 Tables). Furthermore, (PTX±DEX/AZI) led to greater inhibition of R848-induced *TNF* mRNA in newborn compared to adult whole blood (S3 Fig). (PTX±AZI), however, increased LPS- and LPS/ATP-induced *IL10* mRNA to a significantly lesser degree in newborn compared to adult blood (S3 Fig).

PTX and DEX, alone or in combination, inhibited LPS-induced intra-monocytic TNF and IL-1β (Fig 5b), whereas all three anti-inflammatory agents, either alone or in combination, inhibited R848-induced intracellular TNF and IL-1β expression, with greater inhibition of R848-induced intracellular TNF for (PTX+DEX) than for either agent alone (Fig 5c). Consistent with its cAMP-dependent pathway [6], PTX alone increased LPS- and R848-induced intracellular IL-6 in newborn monocytes, whereas DEX and AZI, alone or combined with PTX, inhibited LPS- and/or R848-induced intra-monocytic IL-6 (Fig 5b and 5c). In contrast, none of the tested anti-inflammatory agents inhibited LPS- and R848-induced intracellular IL-10 in newborn monocytes, whereas DEX enhanced R848-induced intracellular IL-10 expression, thus preserving this endogenous anti-inflammatory cytokine (Fig 5b and 5c).

### PTX, DEX and AZI, alone or in combination, inhibited TLR-mediated MAPK phosphorylation in newborn monocytes and increased dual specificity phosphatase 1 (DUSP1) mRNA expression in newborn and adult blood

Whereas only (PTX+DEX) inhibited LPS-induced p38 MAPK phosphorylation in newborn monocytes, PTX, DEX and AZI all inhibited R848-induced p38 MAPK phosphorylation with greater inhibition by (PTX+AZI) compared to either agent alone (S4a Fig). Likewise, (PTX+DEX/AZI) inhibited LPS- and R848-induced JNK phosphorylation, while AZI alone decreased R848-induced JNK phosphorylation (S4b Fig). (DEX±PTX) as well as (PTX+AZI) increased TLR- and/or inflammasome-mediated mRNA expression of *DUSP1*, a key endogenous negative regulator of MAPK phosphorylation (S4c Fig, S5 and S6 Tables).

### Effects of PTX, DEX and AZI, alone or in combination, on nuclear factor κappa B (NF-κB) signaling molecules in newborn and adult blood

Whereas none of the tested anti-inflammatory agents modulated LPS-induced IκBα degradation, (DEX±PTX) as well as (PTX+AZI) modestly diminished R848-induced IκBα degradation in newborn monocytes (S5 Fig). TLR stimulation upregulated *NFKBIA* mRNA expression, which remained unaltered in the presence of these anti-inflammatory agents (S5 and S6 Tables). In contrast, PTX increased TLR- and/or inflammasome-mediated mRNA expression of *NFKB1* and *RELA* in newborn and adult blood, a phenomenon that was not observed in combination with DEX or AZI (S5 and S6 Tables).

### Effects of PTX, DEX and AZI, alone or in combination, on TLR- and/or inflammasome-mediated caspase-1 activation and inflammasome-related gene expression in newborn and adult blood

(PTX±DEX/AZI) inhibited LPS- and R848-induced caspase-1 activation in newborn monocytes, whereby the combinations (PTX+DEX) and (PTX+AZI) exerted greater inhibition of LPS-induced caspase 1 activation than the single agents (S6 Fig). AZI, on the other hand, increased R848- and LPS/ATP-induced caspase-1 activation, which was not observed with (AZI+PTX) (S6 Fig). This increase of LPS/ATP-induced caspase-1 activation in AZI-treated newborn blood monocytes mirrors the observed increase of LPS/ATP-induced IL-1β production in newborn whole blood treated with AZI (Fig 1c, S2 Table). This agent, however, did not exert any effects on TLR- and/or inflammasome-mediated *IL1B* and *CASP1* mRNA expression (S5 and S6 Tables). In contrast, AZI potently inhibited R848-induced IL-1β production in newborn blood (Fig 1c, S2 Table), consistent with AZI’s preferential inhibition of R848-induced cytokines (Table 1).

### (DEX±PTX) increased LPS-induced TLR4 but inhibited R848-induced TLR7 and -8 mRNA and protein expression in newborn and adult blood

Whereas DEX alone increased LPS-induced TLR4 protein expression, PTX and DEX alone or (PTX+DEX) increased LPS-induced *TLR4* mRNA compared to LPS simulation alone (S7 Fig). In contrast, PTX decreased R848-induced TLR7 protein expression, and (PTX+DEX/AZI) inhibited R848-induced *TLR7* mRNA. Likewise, PTX, DEX, or (PTX+DEX/AZI) inhibited R848-induced TLR8 protein expression without altering *TLR8* mRNA in newborn and adult blood (S7 Fig, S5 and S6 Tables). The inhibition of R848-induced TLR7 and -8 expression by (PTX±DEX) is consistent with the observed synergistic inhibition of R848-induced IFN-α production in newborn and adult blood by (PTX+DEX) (Table 2).

## Discussion

Our study was the first to characterize immune-modulatory effects of PTX, DEX, AZI or their combinations on TLR- and/or inflammasome-mediated cytokine production in human newborn and adult whole blood *in vitro*. Neonatal inflammation, which is mediated in part by TLR and inflammasome signaling, can contribute to host defense against infection [33–36]. However, TLR-mediated cytokine induction can also contribute to pathology and disease, including inflammatory diseases of early life such as sepsis, BPD and perinatal brain injury [37–40], which may be modifiable by adjunctive immune-modulatory agents [41]. Due to its anti-TNF activity, PTX has been studied in animals and human adults and newborns to ameliorate inflammatory conditions such as BPD, meconium aspiration, necrotizing enterocolitis, and hypoxic ischemic encephalopathy [7–11], and may decrease all-cause mortality due to its anti-inflammatory activity [12–13]. AZI suppressed the activation of NF-ĸB and the production of pro-inflammatory cytokines [14], shifted human macrophages towards the M2 phenotype, characterized by downregulation of inflammatory and upregulation of anti-inflammatory cytokines [15], and promoted induction of LPS tolerance in mice [16]. A pilot study in human preterm newborns between 24 to 28 weeks gestation given intravenous AZI for 3 days and initiated during the first 3 days of life was efficacious in eradicating *Ureaplasma spp*. from the respiratory tract, and also reduced tracheal aspirate IL-17A concentrations [27]. Thus, the use of these agents is supported by previous reports.

In this study, we employed combination pharmacotherapy, which is an established approach to maximize desired effects while limiting unwanted adverse effects through dose reductions of the individual compounds with distinct activities [17–18]. For example, in murine macrophages studied *in vitro*, whereas PTX inhibited endotoxin-induced cytosolic accumulation of TNF mRNA without affecting the efficiency of mRNA translation, DEX strongly inhibited TNF mRNA translation with only a modest effect on mRNA accumulation, and (PTX+DEX) combination caused greater suppression of TNF biosynthesis than either agent alone [42]. Furthermore, (PTX+DEX) synergistically inhibited LPS-induced lympho-proliferation, expression of intercellular adhesion molecule-1, leukocyte function antigen-1α, and stimuli-induced production of TNF, IL-2, IFN-γ, and IL-12p70 in peripheral blood mononuclear cells from healthy adult donors [43–44]. Except for the Stennert protocol, an anti-phlogistic and rheological therapy for Bell’s palsy [45] and cochlea-vestibular disorders [46], consisting of corticosteroids and infusion of dextran and pentoxifylline, combined PTX and corticosteroids have not been systematically studied. Likewise, we are not aware of any reports studying combination therapy with (PTX+AZI).

Our studies demonstrated potential significance of combination pharmacotherapy in treatment of inflammation. When used as individual anti-inflammatory agents, PTX, DEX, and AZI each demonstrated distinct concentration-dependent inhibition of TLR- and/or inflammasome-mediated cytokine production in human newborn and adult blood. PTX favored a low TNF high IL-6 cytokine production pattern that apparently resembles *in vivo* neonatal inflammatory cytokine responses [47–48]. That PTX preserves and potentially enhances IL-6 production in newborn blood is of particular relevance, since IL-6 on one hand contributes to the protective acute phase response that mobilizes liver-derived host defense factors [49], and, on the other hand, promotes resolution of the innate inflammatory response and development of acquired immune responses [49]. As expected, DEX comparably inhibited the production of all TLR- and/or inflammasome-mediated cytokines except IL-10 [50–51]. Interestingly, low concentration of DEX induced a 3-fold increase in IL-1β production in unstimulated blood, whereas high concentration of DEX suppressed the production of this cytokine. Enhancement of innate immune responses by low concentrations of glucocorticoids, including TLR2 and -4 expression and IL-6, CXCL8 and IL-1 production, through upregulation of DUSP1 and de-phosphorylation of MAPK p38, have previously been reported [52–53].

In contrast, with the exception of IFN-α, produced via TLR7-dependent plasmacytoid dendritic cell activation [54], AZI primarily decreased the production of R848-induced cytokines, especially inflammasome pathway-mediated IL-1β generation. The lack of inhibition of IFN-α production by AZI suggests that it may exert its anti-inflammatory effect via TLR8 and/or inflammasome pathways. As neonatal type I interferon production is weak [33], further suppression of IFN-α production through the actions of anti-inflammatory pharmacological agents may be less desirable. AZI, as opposed to PTX and especially DEX, did not affect R848-induced IFN-α production in newborn cord blood, and may therefore provide an approach to selectively and potently inhibit the pro-inflammatory mediators IL-1β and TNF in the context of infections that engage the TLR8 and inflammasome pathways (eg, influenza and HIV infection), while preserving endogenous antiviral IFN-α responses [55].

Whereas PTX and DEX increased TLR- and/or inflammasome-mediated IL-10 production in adult blood, all three agents decreased the production of this cytokine in newborn samples, albeit to a lesser degree than the production of pro-inflammatory cytokines and thus favoring anti-inflammatory responses in both newborn and adult blood. Newborn blood exhibited greater sensitivity towards the anti-inflammatory actions of PTX compared to adult blood, as evidenced by greater inhibition of TLR- and/or inflammasome-mediated TNF, IL-1β, IL-6 [20], and IFN-α in newborn compared to adult blood. Schüller at al. [56], employing relatively high concentrations of PTX in their *in vitro* experiments (≅ 70, 700, and 7000 μM), also demonstrated similarly marked downregulation by PTX of TNF, IL-1β, IL-6 and IL-10 production in term and preterm human monocytes *in vitro*. As we have previously shown, neonatal plasma factors may contribute to the enhanced anti-inflammatory effects of PTX in newborn blood [20] and neonatal monocytes may be more sensitive to the inhibitory effects of intracellular cAMP [21].

Of note, PTX, DEX, and AZI inhibited LPS- and R848-induced inflammatory cytokine production when added prior to, simultaneously, or after TLRA stimulation, implying that these agents may be suitable as prophylactic and/or therapeutic immune-modulatory agents. As suggested by our experimental findings, treatment of ongoing inflammation may however require higher anti-inflammatory concentrations to achieve the desired effects.

Using a unified and standardized approach to analyze synergistic drug effects [30], we quantified interactions of the anti-inflammatory combinations PTX with DEX or AZI. Our results showed highly synergistic effects of low concentrations of (PTX+DEX) in reducing TLR- and/or inflammasome-mediated TNF, IL-1β, and IL-6 production in human blood. To the extent that these *in vitro* observations hold true *in vivo*, they suggest approaches to exerting steroid-sparing effects. Our findings are in agreement with previous reports, which identified differential inhibition of inflammatory cytokine transcription and translation between PTX and DEX that provided an explanation for their supra-additive anti-inflammatory effects in combination [42–44]. Of relevance, (PTX+DEX) demonstrated significantly higher levels of synergistic inhibition for TLR-mediated TNF production in newborn cord blood compared to adult blood, thus favoring the potential use of this drug combination in neonates. Our study further demonstrated novel and highly synergistic anti-inflammatory effects for low concentration (PTX+AZI) towards suppression of TLR-mediated TNF, IL-1β, and IL-6 production in human blood. PTX and AZI are currently both under study as single adjunct therapeutic agents for newborn sepsis [12–13] and eradication of *Ureaplasma* respiratory tract infection in preterm infants with the secondary outcome of reducing BPD [57]. Furthermore, both agents are apparently relatively safe in neonates when used as individual pharmacological compounds [8,12,27]. In this context and in light of our results, consideration should be given to evaluating (PTX+AZI) combinations as neonatal anti-inflammatory therapies that may enable potent inhibition of inflammation at lower individual doses of each agent. As our data demonstrate, PTX, DEX and AZI show distinct age-dependent individual and synergistic inhibition of inflammation *in vitro*. Although sepsis may promote susceptibility to secondary infections [58], activated leukocytes in septic patients may also contribute to sustained inflammation and organ failure [59]. Infection-induced inflammation in the human newborn may contribute to organ damage [40], suggesting that appropriately formulated and timed anti-inflammatory agents may be of benefit. Such agents may also have clinical application in potential synergistic combination with other adjunctive therapies to support the neonate with bacterial sepsis [60]. Our promising *in vitro* studies now set the stage for a systematic exploration of potential synergy of therapeutic/prophylactic interventions *in vivo*.

PTX, DEX, and AZI, alone or in combination, inhibited LPS- and R848-induced intracellular cytokine production in newborn blood monocytes, mirroring the impact of these agents on TLR-mediated cytokine release into blood culture supernatants. The apparent discrepancy between supernatant IL-10 concentrations and intracellular cytokine staining of newborn monocytes might reflect the contribution of other blood leukocytes, or alterations in cytokine secretion and stability under these experimental conditions.

IL-10 has potent anti-inflammatory properties and contributes to limiting the immune response to infection, thereby preventing damage to the host and maintaining normal tissue homeostasis. PTX and DEX both decreased TLR- and/or inflammasome-mediated *TNF* mRNA and increased the expression of *IL10* mRNA in newborn and adult blood. Importantly, (PTX+DEX) had a greater impact on gene expression compared to either agent alone, mirroring synergistic inhibition of TLR-mediated TNF production by this combination.

Whereas all three agents inhibited R848-induced intracellular IL-1β in newborn blood monocytes, with significantly greater inhibition in (PTX+AZI) combination compared to either agent alone, PTX and DEX, alone or in combination, inhibited LPS-induced intracellular IL-1β. Whereas DEX suppressed LPS- and LPS/ATP-induced *IL1B* mRNA, PTX alone did not affect the gene expression of pro-IL1β, but reduced caspase-1 activation, suggesting a distinct mechanism of inflammasome inhibition. Interestingly, whereas AZI suppressed R848-induced IL-1β in newborn and adult blood and to a lesser degree also LPS-induced IL-1β, it actually increased LPS/ATP-induced production of this cytokine and increased caspase-1 activity in R848- and LPS/ATP-stimulated newborn monocytes. As recently reported, AZI inhibited LPS-induced IL-1α and IL-1β secretion and non-canonical (caspase-4-dependent) inflammasome activation in human monocytes [61]. Likewise, AZI suppressed caspase-1-dependent (canonical) NLRC4- and NLRP3-inflammasome activation in murine macrophages, which was mirrored by inhibition of IL-1β and IL-18 secretion in *Pseudomonas aeruginosa*-infected lungs in mice and humans [62]. These reports are consistent with our findings on AZI-mediated inhibition of LPS-induced IL-1β secretion in newborn and adult blood, whereas the distinct effect on LPS/ATP-induced IL-1β [62] might be due to age-related or experimental differences in the duration and concentration of stimulation conditions. The mechanism by which AZI inhibits R848-induced IL-1β is apparently distinct, as it does not appear to be regulated through inhibition of the caspase-1 pathway and remains to be determined.

Our study features a number of novel aspects including direct benchmarking of several anti-inflammatory agents in human blood *in vitro*, comparison across ages and of combinations. The immune-modulatory effects of PTX and DEX alone have been studied in adult whole blood [51,63–64]. However, to our knowledge, these agents have never before been compared in newborn blood, neither alone nor in combination. In this study, we demonstrated that PTX, DEX, and AZI each inhibited TLR- and/or inflammasome-mediated cytokine production in human newborn and adult blood in a distinct and stimulus-dependent pattern. We took a rigorous statistical approach calculating drug-drug interactions and developing appropriate statistical models. Our results revealed synergistic effects of (PTX+DEX) and (PTX+AZI) on TLR- and/or- inflammasome-mediated pro-inflammatory cytokine production in newborn and adult blood, with greater synergistic anti-inflammatory effects of (PTX+DEX) in newborn compared to adult blood. Finally we characterized the potential mechanisms of the agents studied, providing insight into possible underlying mechanisms involved in the observed synergistic drug effects. Examples are the greater effects of (PTX+DEX) on suppression of *TNF* and enhancement of *IL10* mRNA expression, or the greater inhibition of TLR-mediated MAPK phosphorylation by (PTX+DEX/AZI).

Our study demonstrated several differences in effects of individual and combined anti-inflammatories by age. For example, PTX exerted greater inhibition of inflammatory cytokine production in newborn blood [20] and (PTX±DEX/AZI) led to greater inhibition of R848-induced *TNF* mRNA in newborn compared to adult blood. The combination of PTX and DEX demonstrated greater synergistic inhibition of LPS- and R848-induced TNF production in newborn compared to adult blood. Overall, our study adds to a growing body of work suggesting that drug effects and drug-drug interactions can be age-specific [65]. In an era of precision medicine it becomes important to take ontogeny into account to inform targeted study design and age-optimized treatment regimens.

In addition to the aforementioned strengths, our study also has some limitations. Some of the anti-inflammatory effects demonstrated such as on MAPK phosphorylation and IκB degradation are modest, suggesting that they may not entirely account for the observed effects. Multiple testing adjustment was not implemented due to the explorative nature of the study [31]. The *in vitro* experiments were limited to whole blood from adults and healthy term neonates delivered without evidence of inflammation and in the absence of labor. Future studies should characterize these effects in blood from additional clinically relevant target populations including newborns delivered vaginally in whom labor may influence the inflammatory response, neonates exposed to perinatal infection and inflammation, as well as in preterm neonates. Both the gestational age at delivery of the neonatal study participant and the timing of postnatal sepsis may be key parameters that impact the benefits of anti-inflammatory agents. However, acute inflammatory events in the human newborn may contribute to organ damage, including brain injury [40], suggesting that appropriately formulated and timed anti-inflammatory agents may be of benefit. Future studies should focus on the anti-inflammatory effects of agents such as PTX, DEX and AZI in preterm newborn cord blood and at different postnatal ages *in vitro* as well as on host immunity and organ damage *in vivo*. So far there is no clear evidence of poor neurodevelopmental outcome for infants treated with delayed dexamethasone as a prophylactic treatment after the first week of life, and it remains unclear to date if the benefits of late dexamethasone treatment outweigh the risks in such infants [5,66]. Therefore, current recommendations are that postnatal steroids should be reserved as a rescue therapy for preterm infants which cannot be weaned from mechanical ventilation after the first 7 to 14 days of life [67]. A recent study with low-dose hydrocortisone, showing increased survival without bronchopulmonary dysplasia in preterm infants and without increased neurodevelopmental deficiency at 2 years of age [68–69], suggests that hydrocortisone may also be a promising candidate anti-inflammatory agent for future comparative *in vitro* and *in vivo* studies with pentoxifylline. Furthermore, corticosteroid therapy may have additional therapeutic effects in neonatal sepsis (e.g., for hypotension), although their potential benefit remains controversial and it is not generally recommended for neonatal sepsis [70]. There may be kinetic differences in the PRR-mediated inflammatory responses in newborn blood between LPS, R848 and LPS/ATP, which may have influenced the anti-inflammatory responses to PTX, DEX, and AZI. Future studies employing different durations of PRR-mediated inflammation could clarify these aspects. Since signaling by individual PRR agonists is distinct from stimulation with whole live microbes, e.g., particle size can affect innate immune responses [71] and PRRs such as TLR2 can recognize live bacteria [72], future work should characterize effects in the context of whole live microorganisms. Our *in vitro* whole blood assay system, although replete with relevant cellular and soluble factors important for host responses and having identified agents with subsequent activity *in vivo* [73–74], may not completely reflect responses *in vivo*. Accordingly, the effects of anti-inflammatory combinations of PTX with DEX or AZI should be confirmed *in vivo* employing appropriate neonatal animal models of inflammatory disease such as newborn septic mice, which at the time of birth developmentally correspond to human preterm newborns [75]. Anti-inflammatory agents could be administered either prior to experimental sepsis (prophylactic treatment), at the time of sepsis initiation, or delayed for various lengths of time with respect to the initiation of sepsis (therapeutic treatment), to determine the time period of efficacy for these anti-inflammatory agents. Prompted by promising results of several small studies of PTX in human newborn sepsis suggesting that it may decrease all-cause mortality [12], large-scale multicenter studies of PTX for newborn sepsis are currently underway [76]. Future studies should examine synergistic combinations of these agents that may increase their anti-inflammatory efficacy compared to monotherapy (i.e., improved pharmacokinetics), thereby potentially enhancing therapeutic benefit.

## Conclusions

In summary, our study demonstrated that when tested *in vitro* at clinically relevant concentrations, PTX, DEX and AZI synergistically inhibited LPS-, R848-, and LPS/ATP-induced pro-inflammatory cytokine production in newborn blood. Differential anti-inflammatory mechanisms of the individual agents, including transcriptional and translational regulation, modulation of NF-κB, MAPK, TLR, interferon, and inflammasome pathways, as well as stimulus-specific responses may contribute to the observed synergistic immune-modulatory effects of these drug combinations. In an era of precision medicine, the unique and age-specific anti-inflammatory profiles of PTX, DEX, and AZI could form the basis for more balanced and developmentally adapted anti-inflammatory drug combinations suitable for neonatal clinical use. To the extent that our *in vitro* findings mirror effects of these agents *in vivo*, these combinations may be beneficial in treating perinatal diseases driven by inflammation-mediated pathology. While the anti-inflammatory effects of the agents studied may be beneficial, it should be noted that in those with or at high risk for infection, such agents may be best used with appropriate antimicrobial/antiviral therapy. Future studies, including animal models and clinical trials, should assess the safety and efficacy of these combinations in treating neonatal inflammatory conditions including sepsis *in vivo*.

## Supporting information

S1 Fig Effect of PTX, DEX, and AZI on cytokine production in unstimulated human blood. Newborn cord and adult peripheral blood were treated with (a) PTX (50–200 μM) [S1a Fig adapted from 20, *Speer EM*, *et al*. *Pediatr Res*. *2017;81*: *806–816*], (b) DEX (10^−10^–10^−7^ M), (c) AZI (2.5–20 μM), or vehicle control and cultured for 8 hours at 37°C in 5% CO_2_ in the absence of inflammatory stimulation. Supernatant cytokine concentrations were measured in pg/ml, and median values were plotted against increasing anti-inflammatory drug concentrations. Results from newborn (n = 5) and adult blood (n = 3) were analyzed combined using linear mixed models. Cytokine concentration values were log transformed, in order to meet the assumptions of the model. Correlation among cytokine levels of samples treated with different concentrations of anti-inflammatory agents were modeled as compound symmetry, a covariance structure which assumes that all distinct members of a cluster are equally correlated with each other. Significant differences were indicated: *p<0.05, **p<0.01, *** p<0.001.
(TIF)

S2 Fig Anti-inflammatory agents demonstrate distinct inhibition of TLR- and/or inflammasome-mediated IL-10 and IFN-α production in newborn and adult blood. Newborn cord and adult peripheral blood were pretreated for 2 hours with PTX (50–200 μM), DEX (10^−10^–10^−7^ M), AZI (2.5–20 μM) or vehicle control. Samples were stimulated with 10 ng/ml LPS, 1 μg/ml R848, or LPS followed by 5 mM ATP for 20 min for inflammasome induction, and cultured for 6 hours at 37°C in 5% CO_2_. Supernatant cytokine concentrations were measured, and results (mean ± SEM) expressed as a percentage compared to TLRAs alone, defined as 100%. TLR- and/or inflammasome-mediated production of IL-10 in newborn versus adult blood in response to equimolar (a) PTX, (b) DEX, and (c) AZI. (d) R848-induced IFN-α concentrations in newborn versus adult blood in response to anti-inflammatory agents. The corresponding mean cytokine concentrations in pg/ml for newborn and adult blood induced by TLRAs alone were represented in brackets next to each cohort as part of the graph legends. Significant differences between newborn and adult samples at equimolar anti-inflammatory drug concentrations were indicated: *p<0.05, **p<0.01, ***p<0.001. N = 10 per age group.
(TIF)

S3 Fig PTX exerts greater inhibition of R848-induced *TNF* mRNA and lesser increase in LPS- and LPS/ATP-induced *IL10* mRNA in newborn compared to adult blood. Newborn cord and adult peripheral blood were pretreated for 2 hours with PTX (200 μM), DEX (10^−7^ M), AZI (20 μM) or vehicle control (V), alone or in combination. Samples were stimulated with 10 ng/ml LPS, 1 μg R848, or LPS followed by 5 mM ATP for 20 min for inflammasome induction, and cultured for 2 hours at 37°C in 5% CO_2_. Relative (a) *TNF* mRNA and (b) *IL10* mRNA expression (mean ± SEM) in response to anti-inflammatory agents compared to TLRAs alone, defined as 100%. Wilcoxon rank sum tests were employed to compare gene expression between newborn and adult samples, and significant differences were indicated: *p<0.05, **p<0.01, ***p<0.001. N = 5 per age group.
(TIF)

S4 Fig PTX, DEX, and AZI inhibit TLR-mediated monocytic MAPK phosphorylation and increase DUSP1 mRNA. Whole blood was pretreated for 2 hours with PTX (200 μM), DEX (10^−7^ M), AZI (20 μM) or vehicle control (V), either alone or in combination. Samples were stimulated with 10 ng/ml LPS or 1 μM R848, and cultured for 15 min (flow cytometry) or 1 hour (mRNA expression) at 37°C in 5% CO_2_. MFI of monocytes, gated with forward and side scatter as CD45^+^CD14^+^ cells, was measured as described in *Methods*. Effects of anti-inflammatory treatment on LPS- and R848-induced (a) p38 MAPK and (b) JNK phosphorylation in newborn monocytes (n = 8), plotted as MFI fold changes (± SEM) compared to TLRA stimulation alone (see S6 Data file for raw MFI data). (c) TLR-mediated relative mRNA expression (mean ± SEM) of the *DUSP1* gene in cord and adult blood (n = 5 each, analyzed combined) in response to anti-inflammatory agents compared to TLRA stimulation alone, defined as 100%. Significant differences based on linear mixed models were indicated: *p<0.05, **p<0.01, ***p<0.001.
(TIF)

S5 Fig PTX, DEX and AZI exert modest inhibition of R848-mediated IκBα degradation in newborn monocytes. Whole newborn cord blood (n = 8) was pretreated for 2 hours with PTX (200 μM), DEX (10^−7^ M), AZI (20 μM) or vehicle control (V), either alone or in combination. Samples were stimulated with 10 ng/ml LPS or 1 μM R848, and cultured for 15 min at 37°C in 5% CO_2_. MFI of monocytes, gated with forward and side scatter as CD45^+^CD14^+^ cells, was measured. Inhibitory effects of anti-inflammatory treatment on LPS- and R848-induced total IκBα degradation, plotted as MFI fold changes (± SEM) compared to TLRA stimulation alone (see S6 Data file for raw MFI data). Significant differences based on linear mixed models were indicated: *p<0.05, **p<0.01, ***p<0.001.
(TIF)

S6 Fig PTX±DEX/AZI is the most effective treatment at down-regulating TLR induced caspase-1 activation in newborn monocytes. Whole blood was pretreated for 2 hours with PTX (200 μM), DEX (10^−7^ M), AZI (20 μM) or vehicle control (V), either alone or in combination. Samples were stimulated with 10 ng/ml LPS, 1 μM R848, or LPS followed by 5 mM ATP for 20 min for inflammasome induction, and cultured for 1 hour at 37°C in 5% CO_2_, in the presence of FLICA FAM-YVAD-FMK reagent for flow cytometric caspase-1 activity measurements. Effects of anti-inflammatory treatment on LPS-, R848- and LPS/ATP-induced caspase-1 activation in CD45^+^CD14^+^ newborn monocytes (n = 8), plotted as MFI fold changes (± SEM) compared to TLR- and/or inflammasome-stimulation alone (see S6 Data file for raw MFI data). Significant differences based on linear mixed models were indicated: *p<0.05, **p<0.01, ***p<0.001.
(TIF)

S7 Fig PTX, DEX and (PTX+DEX) decreased R848-induced TLR7 and -8 expression in human blood. Whole blood was pretreated for 2 hours with PTX (200 μM), DEX (10^−7^ M), AZI (20 μM) or vehicle control (V), either alone or in combination. Samples were stimulated with 10 ng/ml LPS or 1 μg R848, and cultured for 1 hour (mRNA expression) or 18 hours (flow cytometry) at 37°C in 5% CO_2_. (a) MFI data from monocytes, gated with forward and side scatter as CD45^+^CD14^+^ cells, were acquired. Effects of anti-inflammatory treatment on LPS- and R848-induced TLR expression in newborn monocytes (n = 8), plotted as MFI fold changes (± SEM) compared to TLRA stimulation alone (see S6 Data file for raw MFI data). (b) Cord and adult blood (n = 5 each, analyzed combined) relative mRNA expression (mean ± SEM) in response to anti-inflammatory agents compared to TLRA alone, defined as 100%. Significant differences based on linear mixed models were indicated: *p<0.05, **p<0.01, ***p<0.001.
(TIF)

S1 Table LPS-, R848-, and LPS/ATP-induced cytokine production in newborn and adult whole blood. (DOCX)

S2 Table PTX, DEX, and AZI inhibited TLR- and/or inflammasome-mediated cytokine production in newborn and adult blood. (DOCX)

S3 Table Converged model fit for hierarchy of synergy models determined by reference agent concentration. (DOCX)

S4 Table Interaction estimates for all converged synergy models in S3 Table. (DOCX)

S5 Table Effects of PTX, DEX and AZI on TLR- and/or inflammasome-induced mRNA expression in newborn and adult blood. (DOCX)

S6 Table Effects of combination treatment vs PTX alone on TLR- and/or inflammasome-induced mRNA expression in newborn and adult blood. (DOCX)

S1 Data file Supernatant cytokine concentrations in unstimulated newborn and adult blood in response to PTX, DEX, or AZI. (XLSX)

S2 Data file TLR- and/or inflammasome-mediated supernatant cytokine concentrations in newborn and adult blood. (XLSX)

S3 Data file TLR- and/or inflammasome-mediated supernatant cytokine concentrations in newborn and adult blood treated with PTX, DEX or AZI. (XLSX)

S4 Data file TLR-mediated cytokine concentrations in newborn and adult blood treated with anti-inflammatory agents prior to, simultaneously or after TLR stimulation. (XLSX)

S5 Data file Effect of combination treatment with (PTX+DEX) vs (PTX+AZI) on TLR- and/or inflammasome-mediated cytokine production in newborn and adult blood. (XLSX)

S6 Data file TLR- and/or inflammasome-mediated intracellular cytokines, caspase-1 activation, MAPK phosphorylation, total IκBα, and TLR expression in cord blood-derived monocytes treated with PTX, DEX, or AZI, alone or in combination. (XLSX)

S7 Data file TLR- and/or inflammasome-mediated gene expression in newborn and adult blood treated with PTX, DEX or AZI, alone or in combination. (XLSX)

S8 Data file Synergistic inhibition of (PTX+DEX) and (PTX+AZI) towards TLR-mediated pro-inflammatory cytokine production in whole blood assays. (XLSX)
