# Supplementary material for: Pentoxifylline, dexamethasone and azithromycin demonstrate distinct age-dependent and synergistic inhibition of TLR- and inflammasome-mediated cytokine production in human newborn and adult blood in vitro
Source: PLoS One. 2018 May 1;13(5):e0196352. doi: 10.1371/journal.pone.0196352 (PMC5929513; doi:10.1371/journal.pone.0196352)
Supplement: S1 Table — (DOCX) [file pone.0196352.s008.docx]

**S1 Table. LPS-, R848-, and LPS/ATP-induced cytokine production in newborn and adult whole blood.**

|  |  | **Newborns** | | **Adults** | |
| --- | --- | --- | --- | --- | --- |
| **TLR agonist** | **Cytokine** | **Mean** | **± STD** | **Mean** | **± STD** |
| LPS | TNF | 3638.4 | 1801.1 | 5454.1 | 1325.0 |
|  | IL-1β | 1571.4 | 668.0 | 3194.3 | 1548.1 |
|  | IL-6 | 33092.2 | 11282.5 | 22938.4 | 5787.7 |
|  | IL-10 | 217.1 | 58.6 | 85.0 | 43.6 |
| R848 | TNF | 23360.0 | 10418.6 | 15711.1 | 5354.0 |
|  | IL-1β | 4346.4 | 1921.4 | 4132.8 | 2463.4 |
|  | IL-6 | 31986.6 | 16810.7 | 21720.1 | 6113.0 |
|  | IL-10 | 721.8 | 241.0 | 407.4 | 121.3 |
|  | IFN-α | 203.4 | 249.3 | 1190.6 | 796.6 |
| LPS/ATP | TNF | 3898.1 | 1261.4 | 5472.5 | 2763.4 |
|  | IL-1β | 13957.4 | 9771.6 | 15599.5 | 6702.4 |
|  | IL-6 | 35470.2 | 21166.2 | 22734.2 | 7273.4 |
|  | IL-10 | 181.3 | 71.3 | 62.6 | 30.9 |

Newborn cord and adult peripheral blood was stimulated with 10 ng/ml LPS, 1 μg/ml R848, or LPS followed by 5 mM ATP for inflammasome induction, and cultured for 6 hours at 37°C in 5% CO_2_. Mean (± STD) supernatant cytokine concentrations in pg/ml for newborn and adult samples. N = 10 per group.
