# Supplementary material for: Pentoxifylline, dexamethasone and azithromycin demonstrate distinct age-dependent and synergistic inhibition of TLR- and inflammasome-mediated cytokine production in human newborn and adult blood in vitro
Source: PLoS One. 2018 May 1;13(5):e0196352. doi: 10.1371/journal.pone.0196352 (PMC5929513; doi:10.1371/journal.pone.0196352)
Supplement: S2 Table — (DOCX) [file pone.0196352.s009.docx]

| **Anti-inflammatory agent** | **Cytokine** | **TLR agonist** | **Increase ↑ or decrease ↓ from baseline (drug concentration)^a^** | | | |
| --- | --- | --- | --- | --- | --- | --- |
|  |  |  | **Newborns** | | **Adults** | |
| PTX | TNF | LPS | **↓** | (≥ 50 µM) | **↓** | (≥ 50 µM) |
|  |  | R848 | **↓** | (≥ 50 µM) | **↓** | (≥ 50 µM) |
|  |  | LPS/ATP | **↓** | (≥ 50 µM) | **↓** | (≥ 50 µM) |
| PTX | IL-1β | LPS | **↓** | (≥ 50 µM) | **↓** | (≥ 50 µM) |
|  |  | R848 | ↓ | (≥ 50 µM) | ↓ | (≥ 50 µM) |
|  |  | LPS/ATP | ↓ | (≥ 50 µM) | ↓ | (≥ 50 µM) |
| PTX | IL-6 | LPS | **↓** | (≥ 50 µM) | **↓** | (≥ 50 µM) |
|  |  | R848 | **↓** | **(≥ 50 µM)** | **↓** | **(400 µM)** |
|  |  | LPS/ATP | **↓** | (≥ 50 µM) | **↓** | (≥ 50 µM) |
| PTX | IL-10 | LPS | **↓** | **(≥ 50 µM)** | **↑** | **(50 – 200 µM)** |
|  |  | R848 | **↓** | **(≥ 100 µM)** | **↓** | **(≥ 200 µM)** |
|  |  | LPS/ATP | **↓** | **(≥ 50 µM)** | **↑** | **(≥ 50 µM)** |
| PTX | IFN-α ^b^ | R848 | **↓** | **(≥ 100 µM)** | **ns** | **---** |
| AZI | TNF | LPS | ns | --- | ns | --- |
|  |  | R848 | **↓** | **(≥ 2.5 µM)** | **↓** | **(≥ 10 µM)** |
|  |  | LPS/ATP | ns | --- | ns | --- |
| AZI | IL-1β | LPS | **↓** | (10 µM) | **↓** | (10 µM) |
|  |  | R848 | **↓** | (≥ 2.5 µM) | **↓** | (≥ 2.5 µM) |
|  |  | LPS/ATP | **↑** | **(10 µM)** | **ns** | **---** |
| AZI | IL-6 | LPS | **ns** | **---** | **↓** | **(20 µM)** |
|  |  | R848 | **↓** | **(≥ 5 µM)** | **↓** | **(≥ 10 µM)** |
|  |  | LPS/ATP | **↓** | (20 µM) | **↓** | (20 µM) |
| AZI | IL-10 | LPS | **↓** | (20 µM) | **↓** | (20 µM) |
|  |  | R848 | **↓** | (≥ 5 µM) | **↓** | (≥ 5 µM) |
|  |  | LPS/ATP | **↓** | **(20 µM)** | **ns** | **---** |
| AZI | IFN-α ^b^ | R848 | ns | --- | ns | --- |
| DEX | TNF | LPS | **↓** | **(≥ 10^-9 M)** | **↓** | **(≥ 10^-8 M)** |
|  |  | R848 | **↓** | (≥ 10^-8 M) | **↓** | (≥ 10^-8 M) |
|  |  | LPS/ATP | **↓** | (≥ 10^-8 M) | **↓** | (≥ 10^-8 M) |
| DEX | IL-1β | LPS | **↓** | (≥ 10^-8 M) | **↓** | (≥ 10^-8 M) |
|  |  | R848 | **↓** | (≥ 10^-8 M) | **↓** | (≥ 10^-8 M) |
|  |  | LPS/ATP | **↓** | (≥ 10^-8 M) | **↓** | (≥ 10^-8 M) |
| DEX | IL-6 | LPS | **↓** | (≥ 10^-8 M) | **↓** | (≥ 10^-8 M) |
|  |  | R848 | **↓** | (≥ 10^-8 M) | **↓** | (≥ 10^-8 M) |
|  |  | LPS/ATP | **↓** | (≥ 10^-8 M) | **↓** | (≥ 10^-8 M) |
| DEX | IL-10 | LPS | **↓** | **(≥ 10^-8 M)** | **↑** | **(≥ 10^-8 M)** |
|  |  | R848 | **↓** | **(10^-7 M)** | **↑** | **(3x10^-8 M)** |
|  |  | LPS/ATP | **↓** | **(≥ 3x10^-8 M)** | **↑** | **(≥ 3x10^-8 M)** |
| DEX | IFN-α ^b^ | R848 | **↓** | **(10^-7 M)** | **↓** | **(≥10^-8 M)** |

Differences in potency of anti-inflammatory agents between newborn and adult samples are indicated in **bold**.

^a^ p-values were based on linear mixed model t-tests.

^b^ LPS- and LPS/ATP-induced IFN-α concentrations were undetectable in most samples.
