# Supplementary material for: Pentoxifylline, dexamethasone and azithromycin demonstrate distinct age-dependent and synergistic inhibition of TLR- and inflammasome-mediated cytokine production in human newborn and adult blood in vitro
Source: PLoS One. 2018 May 1;13(5):e0196352. doi: 10.1371/journal.pone.0196352 (PMC5929513; doi:10.1371/journal.pone.0196352)
Supplement: S3 Table — (DOCX) [file pone.0196352.s010.docx]

S3 Table. Converged model fit for hierarchy of synergy models determined by reference agent concentration.

| **TLR agonist** | **Cytokine** | **Drug combination** | **Reference agent** | **Synergy model** | **Test improvement of model fitting between hierarchical models** | | | | | **Test improvement of model fitting compared to the additive model** | |
| --- | --- | --- | --- | --- | --- | --- | --- | --- | --- | --- | --- |
|  |  |  |  |  | **Residual df** | **Residual sum of squares** | **Test df** | **Test sum of squares** | **p-value^1^** | **Test sum of squares** | **p-value^2^** |
| LPS | TNF | PTX & DEX | DEX [M} | (Null) additive model | 423.00 | 74.74 |  |  |  |  |  |
|  |  |  |  | Common interaction model | 422.00 | 74.47 | 1.00 | 0.28 | 0.2102 | 0.28 | 0.2101 |
| LPS | TNF | PTX & DEX | PTX [uM] | (Null) additive model | 423.00 | 74.74 |  |  |  |  |  |
|  |  |  |  | Common interaction model | 422.00 | 74.47 | 1.00 | 0.28 | 0.2102 | 0.28 | 0.2101 |
|  |  |  |  | Linearly varying interaction values over concentration levels of the reference compound | 421.00 | 63.42 | 1.00 | 11.05 | **<0.001** | 11.33 | **<0.001** |
| LPS | IL-1β | PTX & DEX | DEX [M} | (Null) additive model | 428.00 | 102.18 |  |  |  |  |  |
|  |  |  |  | Common interaction model | 427.00 | 101.56 | 1.00 | 0.61 | 0.1086 | 0.64 | 0.1030 |
| LPS | IL-1β | PTX & DEX | PTX [uM] | (Null) additive model | 428.00 | 102.18 |  |  |  |  |  |
|  |  |  |  | Common interaction model | 427.00 | 101.56 | 1.00 | 0.61 | 0.1086 | 0.64 | 0.1030 |
|  |  |  |  | Linearly varying interaction values over concentration levels of the reference compound | 426.00 | 78.31 | 1.00 | 23.25 | **<0.001** | 23.89 | **<0.001** |
| LPS | IL-6 | PTX & DEX | DEX [M} | (Null) additive model | 248.00 | 60.24 |  |  |  |  |  |
| LPS | IL-6 | PTX & DEX | PTX [uM] | (Null) additive model | 248.00 | 60.24 |  |  |  |  |  |
|  |  |  |  | Linearly varying interaction values over concentration levels of the reference compound | 246.00 | 46.78 | 1.00 | 20.69 | **<0.001** | 13.46 | **<0.001** |
| LPS/ATP | TNF | PTX & DEX | DEX [M} | (Null) additive model | 259.00 | 61.65 |  |  |  |  |  |
|  |  |  |  | Common interaction model | 258.00 | 61.48 | 1.00 | 0.17 | 0.4005 | 0.17 | 0.4005 |
| LPS/ATP | TNF | PTX & DEX | PTX [uM] | (Null) additive model | 259.00 | 61.65 |  |  |  |  |  |
|  |  |  |  | Common interaction model | 258.00 | 61.48 | 1.00 | 0.17 | 0.4005 | 0.17 | 0.4005 |
|  |  |  |  | Linearly varying interaction values over concentration levels of the reference compound | 257.00 | 42.72 | 1.00 | 18.76 | **<0.001** | 18.93 | **<0.001** |
| LPS/ATP | IL-1β | PTX & DEX | DEX [M} | (Null) additive model | 254.00 | 82.66 |  |  |  |  |  |
|  |  |  |  | Common interaction model | 253.00 | 80.91 | 1.00 | 1.75 | **0.020** | 1.75 | **0.020** |
| LPS/ATP | IL-1β | PTX & DEX | PTX [uM] | (Null) additive model | 254.00 | 82.66 |  |  |  |  |  |
|  |  |  |  | Common interaction model | 253.00 | 80.91 | 1.00 | 1.75 | **0.020** | 1.75 | **0.020** |
|  |  |  |  | Linearly varying interaction values over concentration levels of the reference compound | 252.00 | 57.90 | 1.00 | 23.01 | **<0.001** | 24.75 | **<0.001** |
| LPS/ATP | IL-6 | PTX & DEX | DEX [M} | (Null) additive model | 254.00 | 65.83 |  |  |  |  |  |
|  |  |  |  | Common interaction model | 253.00 | 65.70 | 1.00 | 0.13 | 0.4805 | 0.13 | 0.4804 |
| LPS/ATP | IL-6 | PTX & DEX | PTX [uM] | (Null) additive model | 254.00 | 65.83 |  |  |  |  |  |
|  |  |  |  | Common interaction model | 253.00 | 65.70 | 1.00 | 0.13 | 0.4805 | 0.13 | 0.4804 |
|  |  |  |  | Linearly varying interaction values over concentration levels of the reference compound | 252.00 | 50.97 | 1.00 | 14.73 | **<0.001** | 14.86 | **<0.001** |
| R848 | TNF | PTX & DEX | DEX [M} | (Null) additive model | 420.00 | 103.67 |  |  |  |  |  |
|  |  |  |  | Common interaction model | 419.00 | 103.48 | 1.00 | 0.19 | 0.3756 | 0.22 | 0.3443 |
| R848 | TNF | PTX & DEX | PTX [uM] | (Null) additive model | 420.00 | 103.67 |  |  |  |  |  |
|  |  |  |  | Common interaction model | 419.00 | 103.48 | 1.00 | 0.19 | 0.3756 | 0.22 | 0.3443 |
| R848 | IL-1β | PTX & DEX | DEX [M} | (Null) additive model | 414.00 | 98.09 |  |  |  |  |  |
|  |  |  |  | Common interaction model | 413.00 | 97.81 | 1.00 | 0.27 | 0.2836 | 0.27 | 0.2837 |
| R848 | IL-1β | PTX & DEX | PTX [uM] | (Null) additive model | 414.00 | 98.09 |  |  |  |  |  |
|  |  |  |  | Common interaction model | 413.00 | 97.81 | 1.00 | 0.27 | 0.2836 | 0.27 | 0.2837 |
|  |  |  |  | Linearly varying interaction values over concentration levels of the reference compound | 412.00 | 75.56 | 1.00 | 22.25 | **<0.001** | 22.52 | **<0.001** |
| R848 | IL-6 | PTX & DEX | DEX [M} | (Null) additive model | 188.00 | 48.56 |  |  |  |  |  |
|  |  |  |  | Common interaction model | 187.00 | 47.80 | 1.00 | 0.76 | 0.0866 | 0.76 | 0.0866 |
| R848 | IL-6 | PTX & DEX | PTX [uM] | (Null) additive model | 188.00 | 48.56 |  |  |  |  |  |
|  |  |  |  | Common interaction model | 187.00 | 47.80 | 1.00 | 0.76 | 0.0866 | 0.76 | 0.0866 |
| R848 | IFN-α | PTX & DEX | DEX [M} | (Null) additive model | 220.00 | 67.18 |  |  |  |  |  |
|  |  |  |  | Common interaction model | 219.00 | 65.05 | 1.00 | 2.13 | **0.008** | 2.13 | **0.008** |
| R848 | IFN-α | PTX & DEX | PTX [uM] | (Null) additive model | 220.00 | 67.18 |  |  |  |  |  |
|  |  |  |  | Common interaction model | 219.00 | 65.05 | 1.00 | 2.13 | **0.008** | 2.13 | **0.008** |
|  |  |  |  | Linearly varying interaction values over concentration levels of the reference compound | 218.00 | 46.84 | 1.00 | 18.21 | **<0.001** | 20.34 | **<0.001** |
| LPS | TNF | PTX & AZI | AZI [uM] | (Null) additive model | 209.00 | 32.45 |  |  |  |  |  |
|  |  |  |  | Common interaction model | 208.00 | 31.05 | 1.00 | 1.40 | **0.003** | 1.40 | **0.003** |
|  |  |  |  | Separately varying interaction values over each concentration level of the reference agent | 205.00 | 21.35 | 2.00 | 1.85 | **0.002** | 11.09 | **<0.001** |
|  |  |  |  | Separate interaction values for each concentration combination | 193.00 | 14.20 | 12.00 | 7.15 | **<0.001** | 18.24 | **<0.001** |
| LPS | TNF | PTX & AZI | PTX [uM] | (Null) additive model | 209.00 | 32.45 |  |  |  |  |  |
|  |  |  |  | Common interaction model | 208.00 | 31.05 | 1.00 | 1.40 | **0.003** | 1.40 | **0.003** |
|  |  |  |  | Separate interaction values for each concentration combination | 193.00 | 14.20 | 13.00 | 9.36 | **<0.001** | 18.24 | **<0.001** |
| LPS | IL-1β | PTX & AZI | AZI [uM] | (Null) additive model | 220.00 | 49.41 |  |  |  |  |  |
|  |  |  |  | Common interaction model | 219.00 | 47.14 | 1.00 | 2.27 | **0.001** | 2.27 | **0.001** |
|  |  |  |  | Linearly varying interaction values over concentration levels of the reference compound | 218.00 | 39.05 | 1.00 | 8.09 | **<0.001** | 10.36 | **<0.001** |
| LPS | IL-1β | PTX & AZI | PTX [uM] | (Null) additive model | 220.00 | 49.41 |  |  |  |  |  |
|  |  |  |  | Common interaction model | 219.00 | 47.14 | 1.00 | 2.27 | **0.001** | 2.27 | **0.001** |
| LPS | IL-6 | PTX & AZI | AZI [uM] | (Null) additive model | 203.00 | 50.42 | . |  |  |  |  |
|  |  |  |  | Common interaction model | 202.00 | 46.28 | 1.00 | 4.14 | **<0.001** | 4.14 | **<0.001** |
|  |  |  |  | Separately varying interaction values over each concentration level of the reference agent | 199.00 | 32.77 | 3.00 | 1.98 | **0.009** | 17.65 | **<0.001** |
| LPS | IL-6 | PTX & AZI | PTX [uM] | (Null) additive model | 203.00 | 50.42 |  |  |  |  |  |
|  |  |  |  | Common interaction model | 202.00 | 46.28 | 1.00 | 4.14 | **<0.001** | 4.14 | **<0.001** |
| LPS/ATP | TNF | PTX & AZI | AZI [uM] | (Null) additive model | 221.00 | 35.52 |  |  |  |  |  |
|  |  |  |  | Common interaction model | 220.00 | 33.66 | 1.00 | 1.86 | **<0.001** | 1.86 | **<0.001** |
|  |  |  |  | Separately varying interaction values over each concentration level of the reference agent | 217.00 | 22.59 | 2.00 | 113.35 | **<0.001** | 12.92 | **<0.001** |
|  |  |  |  | Separate interaction values for each concentration combination | 205.00 | 17.46 | 12.00 | 5.14 | **<0.001** | 18.06 | **<0.001** |
| LPS/ATP | TNF | PTX & AZI | PTX [uM] | (Null) additive model | 221.00 | 35.52 |  |  |  |  |  |
|  |  |  |  | Common interaction model | 220.00 | 33.66 | 1.00 | 1.86 | **<0.001** | 1.86 | **<0.001** |
|  |  |  |  | Separate interaction values for each concentration combination | 205.00 | 17.46 | 13.00 | 12.62 | **<0.001** | 18.06 | **<0.001** |
| LPS/ATP | IL-1β | PTX & AZI | AZI [uM] | (Null) additive model | 182.00 | 48.14 |  |  |  |  |  |
|  |  |  |  | Common interaction model | 181.00 | 44.33 | 1.00 | 3.81 | **0.001** | 3.81 | **0.001** |
|  |  |  |  | Separately varying interaction values over each concentration level of the reference agent | 178.00 | 32.55 | 2.00 | 1.34 | **0.027** | 15.59 | **<0.001** |
|  |  |  |  | Linearly varying interaction values over concentration levels of the reference compound | 180.00 | 33.89 | 1.00 | 10.44 | **<0.001** | 14.25 | **<0.001** |
| LPS/ATP | IL-1β | PTX & AZI | PTX [uM] | (Null) additive model | 182.00 | 48.14 |  |  |  |  |  |
|  |  |  |  | Common interaction model | 181.00 | 44.33 | 1.00 | 3.81 | **0.001** | 3.81 | **0.001** |
| LPS/ATP | IL-6 | PTX & AZI | AZI [uM] | (Null) additive model | 213.00 | 42.44 |  |  |  |  |  |
|  |  |  |  | Common interaction model | 212.00 | 40.51 | 1.00 | 1.93 | **0.002** | 1.93 | **0.002** |
|  |  |  |  | Separately varying interaction values over each concentration level of the reference agent | 209.00 | 29.59 | 2.00 | 2.25 | **<0.001** | 12.84 | **<0.001** |
|  |  |  |  | Separate interaction values for each concentration combination | 197.00 | 17.21 | 12.00 | 12.39 | **<0.001** | 25.23 | **<0.001** |
| LPS/ATP | IL-6 | PTX & AZI | PTX [uM] | (Null) additive model | 213.00 | 42.44 |  |  |  |  |  |
|  |  |  |  | Common interaction model | 212.00 | 40.51 | 1.00 | 1.93 | **0.002** | 1.93 | **0.002** |
|  |  |  |  | Separate interaction values for each concentration combination | 197.00 | 17.21 | 13.00 | 10.94 | **<0.001** | 25.23 | **<0.001** |
| R848 | TNF | PTX & AZI | AZI [uM] | (Null) additive model | 232.00 | 41.25 |  |  |  |  |  |
|  |  |  |  | Common interaction model | 231.00 | 39.46 | 1.00 | 1.80 | **0.001** | 1.80 | **0.001** |
|  |  |  |  | Linearly varying interaction values over concentration levels of the reference compound | 230.00 | 34.36 | 1.00 | 5.09 | **<0.001** | 6.89 | **<0.001** |
| R848 | TNF | PTX & AZI | PTX [uM] | (Null) additive model | 232.00 | 41.25 |  |  |  |  |  |
|  |  |  |  | Common interaction model | 231.00 | 39.46 | 1.00 | 1.80 | **0.001** | 1.80 | **0.001** |
| R848 | IL-1β | PTX & AZI | AZI [uM] | (Null) additive model | 232.00 | 46.90 |  |  |  |  |  |
|  |  |  |  | Common interaction model | 231.00 | 44.89 | 1.00 | 2.01 | **0.002** | 2.01 | **0.002** |
|  |  |  |  | Linearly varying interaction values over concentration levels of the reference compound | 230.00 | 36.85 | 1.00 | 8.03 | **<0.001** | 10.05 | **<0.001** |
| R848 | IL-1β | PTX & AZI | PTX [uM] | (Null) additive model | 232.00 | 46.90 |  |  |  |  |  |
|  |  |  |  | Common interaction model | 231.00 | 44.89 | 1.00 | 2.01 | **0.002** | 2.01 | **0.002** |
| R848 | IL-6 | PTX & AZI | AZI [uM] | (Null) additive model | 183.00 | 34.23 |  |  |  |  |  |
|  |  |  |  | Common interaction model | 182.00 | 32.87 | 1.00 | 1.37 | **0.007** | 1.37 | **0.007** |
|  |  |  |  | Linearly varying interaction values over concentration levels of the reference compound | 181.00 | 28.09 | 1.00 | 4.77 | **<0.001** | 6.14 | **<0.001** |
| R848 | IL-6 | PTX & AZI | PTX [uM] | (Null) additive model | 183.00 | 34.23 |  |  |  |  |  |
|  |  |  |  | Common interaction model | 182.00 | 32.87 | 1.00 | 1.37 | **0.007** | 1.37 | **0.007** |

The determination of drug interactions for combined anti-inflammatory treatment effects were based on Loewe’s definition of additivity [29] and determined as described by Harbron [30], by calculating a hierarchy of interaction indices to best fit the synergistic model. A common model across all concentration combinations, a model of linearly varying values over concentration levels of one of the compounds, a model of separately varying values for each concentration level of one of the compounds, and a model of separate values for each concentration combination were fitted for each drug combination and compared to the additive model. To evaluate the performance between models, a set of hierarchical goodness of fit tests were applied. Significant p-values are presented in bold. PTX (50 to 400 µM) and AZI (2.5 to 20 µM) each had 4 different concentration levels, while DEX (10^-10^ to 10^-7^ M) had 5 concentration levels. Each drug combination dataset included cytokine concentration values measured in whole blood culture supernatants from 10 subjects (5 newborns and 5 adults; 8 newborns and 8 adults for LPS- and R848-induced TNF and IL-1β treated with combined PTX and DEX). The drug synergy determinations were based on results from all subjects combined. Negative values of drug effects, i.e. cytokine concentrations of treated samples were above those of untreated samples, were removed. The SAS NLIN procedure was used to fit the models. The table showed the converged model fit. For R848-induced IFN-α samples treated with PTX and AZI no model converged.

^1,2^ p-value was based on F-test.
