# Supplementary material for: Pentoxifylline, dexamethasone and azithromycin demonstrate distinct age-dependent and synergistic inhibition of TLR- and inflammasome-mediated cytokine production in human newborn and adult blood in vitro
Source: PLoS One. 2018 May 1;13(5):e0196352. doi: 10.1371/journal.pone.0196352 (PMC5929513; doi:10.1371/journal.pone.0196352)
Supplement: S4 Table — (DOCX) [file pone.0196352.s011.docx]

S4 Table. Interaction estimates for all converged synergy models in S3 Table.

| **TLR agonist** | **Cytokine** | **Drug combination** | **Reference agent** | **Synergy model** | **Drug concentrations** | **Interaction index (95% CI)** | **p-value^1^** |
| --- | --- | --- | --- | --- | --- | --- | --- |
| LPS | TNF | PTX & DEX | PTX | Common interaction model |  | 0.93 (0.83-1.04) | 0.213 |
|  |  |  |  | Linearly varying interaction values over concentration levels of the reference compound | **50 µM PTX** | **0.55 (0.47-0.63)** | **<0.001** |
|  |  |  |  |  | **100 µM PTX** | **0.78 (0.71-0.86)** | **<0.001** |
|  |  |  |  |  | 200 µM PTX | 1.01 (0.91-1.12) | 0.791 |
|  |  |  |  |  | 400 µM PTX | 1.25 (1.09-1.40) | 0.002 |
| LPS | IL-1β | PTX & DEX | PTX | Common interaction model |  | 1.12 (0.98-1.27) | 0.100 |
|  |  |  |  | Linearly varying interaction values over concentration levels of the reference compound | **50 µM PTX** | **0.50 (0.42-0.57)** | **<0.001** |
|  |  |  |  |  | **100 µM PTX** | **0.80 (0.71-0.90)** | **<0.001** |
|  |  |  |  |  | 200 µM PTX | 1.11 (0.98-1.25) | 0.093 |
|  |  |  |  |  | 400 µM PTX | 1.42 (1.24-1.61) | <0.001 |
| LPS | IL-6 | PTX & DEX | PTX | Linearly varying interaction values over concentration levels of the reference compound | **50 µM PTX** | **0.39 (0.29-0.48)** | **<0.001** |
|  |  |  |  |  | **100 µM PTX** | **0.69 (0.58-0.81)** | **<0.001** |
|  |  |  |  |  | 200 µM PTX | 1.00 (0.84-1.16) | 0.982 |
|  |  |  |  |  | 400 µM PTX | 1.30 (1.08-1.52) | 0.007 |
| LPS/ATP | TNF | PTX & DEX | PTX | Common interaction model |  | 0.93 (0.78-1.10) | 0.398 |
|  |  |  |  | Linearly varying interaction values over concentration levels of the reference compound | **50 µM PTX** | **0.36 (0.29-0.43)** | **<0.001** |
|  |  |  |  |  | **100 µM PTX** | **0.66 (0.57-0.76)** | **<0.001** |
|  |  |  |  |  | 200 µM PTX | 0.97 (0.83-1.11) | 0.672 |
|  |  |  |  |  | 400 µM PTX | 1.28 (1.07-1.48) | 0.007 |
| LPS-ATP | IL-1β | PTX & DEX | PTX | Common interaction model |  | 1.30 (1.06-1.60) | 0.013 |
|  |  |  |  | Linearly varying interaction values over concentration levels of the reference compound | **50 µM PTX** | **0.22 (0.17-0.28)** | **<0.001** |
|  |  |  |  |  | **100 µM PTX** | **0.56 (0.45-0.68)** | **<0.001** |
|  |  |  |  |  | 200 µM PTX | 0.90 (0.72-1.08) | 0.282 |
|  |  |  |  |  | 400 µM PTX | 1.24 (0.98-1.49) | 0.071 |
| LPS/ATP | IL-6 | PTX & DEX | PTX | Common interaction model |  | 1.07 (0.89-1.27) | 0.475 |
|  |  |  |  | Linearly varying interaction values over concentration levels of the reference compound | **50 µM PTX** | **0.37 (0.28-0.46)** | **<0.001** |
|  |  |  |  |  | **100 µM PTX** | **0.67 (0.56-0.79)** | **<0.001** |
|  |  |  |  |  | 200 µM PTX | 0.98 (0.82-1.14) | 0.804 |
|  |  |  |  |  | 400 µM PTX | 1.28 (1.06-1.51) | 0.014 |
| R848 | TNF | PTX & DEX | PTX | Common interaction model |  | 1.06 (0.93-1.21) | 0.370 |
| R848 | IL-1β | PTX & DEX | PTX | Common interaction model |  | 1.08 (0.94-1.23) | 0.274 |
|  |  |  |  | Linearly varying interaction values over concentration levels of the reference compound | **50 µM PTX** | **0.43 (0.35-0.50)** | **<0.001** |
|  |  |  |  |  | **100 µM PTX** | **0.74 (0.65-0.83)** | **<0.001** |
|  |  |  |  |  | 200 µM PTX | 1.05 (0.93-1.18) | 0.413 |
|  |  |  |  |  | 400 µM PTX | 1.37 (1.19-1.54) | <0.001 |
| R848 | IL-6 | PTX & DEX | PTX | Common interaction model |  | 1.21 (0.99-1.49) | 0.068 |
| R848 | IFN-α | PTX & DEX | PTX | Common interaction model |  | 1.35 (1.10-1.65) | 0.004 |
|  |  |  |  | Linearly varying interaction values over concentration levels of the reference compound | **50 µM PTX** | **0.31 (0.22-0.39)** | **<0.001** |
|  |  |  |  |  | **100 µM PTX** | **0.66 (0.53-0.79)** | **<0.001** |
|  |  |  |  |  | 200 µM PTX | 1.01 (0.82-1.21) | 0.888 |
|  |  |  |  |  | 400 µM PTX | 1.37 (1.10-1.64) | 0.008 |
| LPS | TNF | PTX & AZI | AZI | Common interaction model |  | 1.30 (1.10-1.53) | 0.002 |
|  |  |  |  | Separately varying interaction values over each concentration level of the reference agent | 2.5 µM AZI | 1.11 (0.95-1.30) | 0.199 |
|  |  |  |  |  | 5 µM AZI | 1.36 (1.15-1.62) | <0.001 |
|  |  |  |  |  | 10 µM AZI | 1.90 (1.56-2.32) | <0.001 |
|  |  |  |  |  | 20 µM AZI | 3.02 (2.40-3.80) | <0.001 |
|  |  |  |  | Separate interaction values for each concentration combination | **50 µM PTX & 2.5 µM AZI** | **0.72 (0.58,0.89)** | **0.003** |
|  |  |  |  |  | 50 µM PTX & 5 µM AZI | 0.96 (0.78-1.20) | 0.743 |
|  |  |  |  |  | 50 µM PTX & 10 µM AZI | 1.51 (1.20-1.90) | <0.001 |
|  |  |  |  |  | 50 µM PTX & 20 µM AZI | 2.60 (2.05-3.30) | <0.001 |
|  |  |  |  |  | 100 µM PTX & 2.5 µM AZI | 0.96 (0.78-1.19) | 0.726 |
|  |  |  |  |  | 100 µM PTX & 5 µM AZI | 1.31 (1.05-1.63) | 0.018 |
|  |  |  |  |  | 100 µM PTX & 10 µM AZI | 1.96 (1.54-2.48) | <0.001 |
|  |  |  |  |  | 100 µM PTX & 20 µM AZI | 3.26 (2.53-4.19) | <0.001 |
|  |  |  |  |  | 200 µM PTX & 2.5 µM AZI | 1.32 (1.08-1.63) | 0.008 |
|  |  |  |  |  | 200 µM PTX & 5 µM AZI | 1.72 (1.37-2.16) | <0.001 |
|  |  |  |  |  | 200 µM PTX & 10 µM AZI | 2.53 (1.96-3.27) | <0.001 |
|  |  |  |  |  | 200 µM PTX & 20 µM AZI | 4.25 (3.17-5.70) | <0.001 |
|  |  |  |  |  | 400 µM PTX & 2.5 µM AZI | 1.79 (1.45-2.21) | <0.001 |
|  |  |  |  |  | 400 µM PTX & 5 µM AZI | 2.28 (1.80-2.90) | <0.001 |
|  |  |  |  |  | 400 µM PTX & 10 µM AZI | 3.31 (2.49-4.38) | <0.001 |
|  |  |  |  |  | 400 µM PTX & 20 µM AZI | 5.35 (3.90-7.35) | <0.001 |
| LPS | IL-1β | PTX & AZI | AZI | Common interaction model |  | 1.35 (1.14-1.60) | <0.001 |
|  |  |  |  | Linearly varying interaction values over concentration levels of the reference compound | **2.5 µM AZI** | **0.80 (0.63-0.97)** | **0.020** |
|  |  |  |  |  | 5 µM AZI | 1.12 (0.93-1.30) | 0.211 |
|  |  |  |  |  | 10 µM AZI | 1.44 (1.18-1.69) | <0.001 |
|  |  |  |  |  | 20 µM AZI | 1.75 (1.41-2.10) | <0.001 |
| LPS | IL-6 | PTX & AZI | AZI | Common interaction model |  | 1.56 (1.30-1.88) | <0.001 |
|  |  |  |  | Separately varying interaction values over each concentration level of the reference agent | 2.5 µM AZI | 0.82 (0.66-1.02) | 0.073 |
|  |  |  |  |  | 5 µM AZI | 1.01 (0.82-1.24) | 0.917 |
|  |  |  |  |  | 10 µM AZI | 1.47 (1.20-1.80) | <0.001 |
|  |  |  |  |  | 20 µM AZI | 2.36 (1.91-2.91) | <0.001 |
| LPS/ATP | TNF | PTX & AZI | AZI | Common interaction model |  | 1.36 (1.15,1.60) | <0.001 |
|  |  |  |  | Separately varying interaction values over each concentration level of the reference agent | 2.5 µM AZI | 1.16 (0.99,1.36) | 0.059 |
|  |  |  |  |  | 5 µM AZI | 1.42 (1.20,1.68) | <0.001 |
|  |  |  |  |  | 10 µM AZI | 2.00 (1.65,2.43) | <0.001 |
|  |  |  |  |  | 20 µM AZI | 3.22 (2.59,4.01) | <0.001 |
|  |  |  |  | Separate interaction values for each concentration combination | **50µM PTX & 2.5 µM AZI** | **0.77 (0.60-0.98)** | **0.033** |
|  |  |  |  |  | 50 µM PTX & 5 µM AZI | 1.02 (0.81-1.30) | 0.859 |
|  |  |  |  |  | 50 µM PTX & 10 µM AZI | 1.55 (1.21-1.97) | <0.001 |
|  |  |  |  |  | 50 µM PTX & 20 µM AZI | 2.62 (2.05-3.35) | <0.001 |
|  |  |  |  |  | 100 µM PTX & 2.5 µM AZI | 1.00 (0.79-1.25) | 0.974 |
|  |  |  |  |  | 100 µM PTX & 5 µM AZI | 1.30 (1.02-1.64) | 0.031 |
|  |  |  |  |  | 100 µM PTX & 10 µM AZI | 1.91 (1.49-2.46) | <0.001 |
|  |  |  |  |  | 100 µM PTX & 20 µM AZI | 3.16 (2.43-4.13) | <0.001 |
|  |  |  |  |  | 200 µM PTX & 2.5 µM AZI | 1.31 (1.06-1.64) | 0.015 |
|  |  |  |  |  | 200 µM PTX & 5 µM AZI | 1.65 (1.30-2.09) | <0.001 |
|  |  |  |  |  | 200 µM PTX & 10 µM AZI | 2.40 (1.84-3.13) | <0.001 |
|  |  |  |  |  | 200 µM PTX & 20 µM AZI | 3.89 (2.90-5.22) | <0.001 |
|  |  |  |  |  | 400 µM PTX & 2.5 µM AZI | 1.66 (1.33-2.08) | <0.001 |
|  |  |  |  |  | 400 µM PTX & 5 µM AZI | 2.08 (1.63-2.66) | <0.001 |
|  |  |  |  |  | 400 µM PTX & 10 µM AZI | 2.98 (2.25-3.96) | <0.001 |
|  |  |  |  |  | 400 µM PTX & 20 µM AZI | 4.77 (3.48-6.54) | <0.001 |
| LPS/ATP | IL-1β | PTX & AZI | AZI | Common interaction model |  | 1.58 (1.28-1.94) | <0.001 |
|  |  |  |  | Separately varying interaction values over each concentration level of the reference agent | 2.5 µM AZI | 0.85 (0.67-1.08) | 0.178 |
|  |  |  |  |  | 5 µM AZI | 1.15 (0.92-1.44) | 0.214 |
|  |  |  |  |  | 10 µM AZI | 1.62 (1.29-2.03) | <0.001 |
|  |  |  |  |  | 20 µM AZI | 2.63 (2.04-3.39) | <0.001 |
|  |  |  |  | Linearly varying interaction values over concentration levels of the reference compound | 2.5 µM AZI | 0.83 (0.64-1.02) | 0.080 |
|  |  |  |  |  | 5 µM AZI | 1.31 (1.07-1.55) | 0.012 |
|  |  |  |  |  | 10 µM AZI | 1.79 (1.43-2.14) | <0.001 |
|  |  |  |  |  | 20 µM AZI | 2.27 (1.78-2.75) | <0.001 |
| LPS/ATP | IL-6 | PTX & AZI | AZI | Common interaction model |  | 1.35 (1.13-1.62) | <0.001 |
|  |  |  |  | Separately varying interaction values over each concentration level of the reference agent | 2.5 µM AZI | 0.93 (0.76-1.12) | 0.432 |
|  |  |  |  |  | 5 µM AZI | 1.06 (0.88-1.28) | 0.505 |
|  |  |  |  |  | 10 µM AZI | 1.46 (1.19-1.78) | <0.001 |
|  |  |  |  |  | 20 µM AZI | 2.33 (1.86-2.91) | <0.001 |
|  |  |  |  | Separate interaction values for each concentration combination | **50µM PTX & 2.5 µM AZI** | **0.40 (0.31-0.51)** | **<0.001** |
|  |  |  |  |  | **50 µM PTX & 5 µM AZI** | **0.55 (0.44-0.69)** | **<0.001** |
|  |  |  |  |  | 50 µM PTX & 10 µM AZI | 0.87 (0.69-1.10) | 0.247 |
|  |  |  |  |  | 50 µM PTX & 20 µM AZI | 1.54 (1.19-1.98) | <0.001 |
|  |  |  |  |  | **100 µM PTX & 2.5 µM AZI** | **0.57 (0.46-0.71)** | **<0.001** |
|  |  |  |  |  | **100 µM PTX & 5 µM AZI** | **0.73 (0.59-0.91)** | **0.006** |
|  |  |  |  |  | 100 µM PTX & 10 µM AZI | 1.05 (0.83-1.33) | 0.679 |
|  |  |  |  |  | 100 µM PTX & 20 µM AZI | 1.72 (1.33-2.23) | <0.001 |
|  |  |  |  |  | 200 µM PTX & 2.5 µM AZI | 0.88 (0.72-1.09) | 0.249 |
|  |  |  |  |  | 200 µM PTX & 5 µM AZI | 1.05 (0.85-1.30) | 0.665 |
|  |  |  |  |  | 200 µM PTX & 10 µM AZI | 1.38 (1.10-1.73) | 0.006 |
|  |  |  |  |  | 200 µM PTX & 20 µM AZI | 2.06 (1.60-2.65) | <0.001 |
|  |  |  |  |  | 400 µM PTX & 2.5 µM AZI | 1.52 (1.23-1.89) | <0.001 |
|  |  |  |  |  | 400 µM PTX & 5 µM AZI | 1.68 (1.35-2.07) | <0.001 |
|  |  |  |  |  | 400 µM PTX & 10 µM AZI | 2.03 (1.63-2.52) | <0.001 |
|  |  |  |  |  | 400 µM PTX & 20 µM AZI | 2.73 (2.16-3.44) | <0.001 |
| R848 | TNF | PTX & AZI | AZI | Common interaction model |  | 1.27 (1.11-1.46) | <0.001 |
|  |  |  |  | Linearly varying interaction values over concentration levels of the reference compound | 2.5 µM AZI | 0.91 (0.76-1.06) | 0.247 |
|  |  |  |  |  | 5 µM AZI | 1.17 (1.02-1.32) | 0.030 |
|  |  |  |  |  | 10 µM AZI | 1.43 (1.23-1.63) | <0.001 |
|  |  |  |  |  | 20 µM AZI | 1.68 (1.41-1.96) | <0.001 |
| R848 | IL-1β | PTX & AZI | AZI | Common interaction model |  | 1.29 (1.11-1.50) | <0.001 |
|  |  |  |  | Linearly varying interaction values over concentration levels of the reference compound | **2.5 µM AZI** | **0.79 (0.64-0.93)** | **0.004** |
|  |  |  |  |  | 5 µM AZI | 1.08 (0.93-1.22) | 0.289 |
|  |  |  |  |  | 10 µM AZI | 1.37 (1.18-1.56) | <0.001 |
|  |  |  |  |  | 20 µM AZI | 1.66 (1.40-1.92) | <0.001 |
| R848 | IL-6 | PTX & AZI | AZI | Common interaction model |  | 1.28 (1.08-1.52) | 0.004 |
|  |  |  |  | Linearly varying interaction values over concentration levels of the reference compound | **2.5 µM AZI** | **0.81 (0.63-0.99)** | **0.035** |
|  |  |  |  |  | 5 µM AZI | 1.07 (0.90-1.24) | 0.428 |
|  |  |  |  |  | 10 µM AZI | 1.33 (1.12-1.54) | 0.003 |
|  |  |  |  |  | 20 µM AZI | 1.58 (1.31-1.86) | <0.001 |

The table showed the estimates for interaction indices along with 95% CIs for the converged model fits from S3 Table. Interaction indices below 1 indicated synergy, with lower values representing higher levels of synergy. Significant synergistic anti-inflammatory combinations are presented in **bold**.

^1^ p-value was based on t-test.
