# Supplementary material for: Pentoxifylline, dexamethasone and azithromycin demonstrate distinct age-dependent and synergistic inhibition of TLR- and inflammasome-mediated cytokine production in human newborn and adult blood in vitro
Source: PLoS One. 2018 May 1;13(5):e0196352. doi: 10.1371/journal.pone.0196352 (PMC5929513; doi:10.1371/journal.pone.0196352)
Supplement: S5 Table — (DOCX) [file pone.0196352.s012.docx]

S5 Table. Effects of PTX, DEX and AZI on TLR- and/or inflammasome-induced mRNA expression in newborn and adult blood.

| Gene | TLR agonist | Duration of stimulation | mRNA expression changes to TLR agonist stimulation (Mean ΔΔCT) ^b^ | | | | | | | | | | | | |
| --- | --- | --- | --- | --- | --- | --- | --- | --- | --- | --- | --- | --- | --- | --- | --- |
|  |  |  | CTL ^a^ | | PTX ^a^ | | DEX | | AZI | | PTX+DEX | | | PTX+AZI | |
| *CASP1* | LPS | 1H | ↑ | -0.32 | ↑↑ | -0.41 | ∅ | -0.14 | ∅ | -0.26 | ∅ | | -0.03 | ∅ | 0.05 |
|  | R848 | 1H | ∅ | -0.05 | ∅ | -0.31 | ∅ | -0.27 | ∅ | -0.10 | ∅ | | 0.10 | ∅ | -0.08 |
|  | LPS/ATP | 1H | ↑↑↑ | -0.68 | ↑ | -0.40 | ∅ | -0.23 | ∅ | -0.15 | ∅ | | -0.23 | ∅ | -0.14 |
|  | LPS | 2H | ∅ | 0.21 | ∅ | -0.23 | ∅ | -0.16 | ∅ | -0.18 | ∅ | | -0.10 | ∅ | -0.00 |
|  | R848 | 2H | ∅ | 0.15 | ↑↑ | -0.42 | ∅ | -0.27 | ∅ | -0.21 | ∅ | | -0.17 | ↑ | -0.31 |
|  | LPS/ATP | 2H | ∅ | -0.09 | ∅ | -0.30 | ∅ | -0.23 | ∅ | 0.14 | ∅ | | -0.22 | ∅ | -0.05 |
| *DUSP1* | LPS | 1H | ∅ | 0.11 | ∅ | -0.08 | ∅ | -0.50 | ∅ | -0.19 | ↑ | | -0.62 | ∅ | -0.36 |
|  | R848 | 1H | ∅ | 0.19 | ∅ | -0.22 | ↑↑ | -0.53 | ∅ | -0.09 | ↑↑↑ | | -0.68 | ↑ | -0.43 |
|  | LPS/ATP | 1H | ∅ | 0.10 | ↑ | -0.39 | ↑↑ | -0.57 | ∅ | -0.25 | ↑↑↑ | | -0.79 | ↑ | -0.41 |
|  | LPS | 2H | ∅ | -0.06 | ∅ | -0.28 | ∅ | -0.46 | ∅ | 0.20 | ↑↑ | | -0.82 | ∅ | -0.29 |
|  | R848 | 2H | ∅ | -0.17 | ↑ | -0.44 | ↑↑↑ | -0.85 | ∅ | 0.17 | ↑↑↑ | | -1.10 | ∅ | -0.34 |
|  | LPS/ATP | 2H | ∅ | 0.24 | ∅ | -0.36 | ↑ | -0.42 | ∅ | 0.02 | ↑ | | -0.50 | ∅ | -0.29 |
| *IL10* | LPS | 1H | ∅ | -0.37 | ↑↑↑ | -1.11 | ↑↑↑ | -1.43 | ∅ | -0.16 | ↑↑↑ | | -2.36 | ↑↑↑ | -1.45 |
|  | R848 | 1H | ∅ | -0.69 | ∅ | -0.84 | ↑↑↑ | -1.62 | ∅ | -0.27 | ↑↑↑ | | -2.31 | ↑↑ | -1.27 |
|  | LPS/ATP | 1H | ∅ | -0.09 | ↑↑↑ | -1.37 | ↑↑↑ | -1.25 | ∅ | -0.25 | ↑↑↑ | | -2.41 | ↑↑↑ | -1.09 |
|  | LPS | 2H | ↓↓↓ | 1.14 | ↑↑ | -0.89 | ↑↑↑ | -2.36 | ∅ | -0.23 | ↑↑↑ | -2.43 | | ↑ | -0.69 |
|  | R848 | 2H | ↓↓↓ | 1.60 | ∅ | 0.06 | ↑↑ | -1.41 | ∅ | 0.09 | ↑↑↑ | -1.76 | | ∅ | -0.30 |
|  | LPS/ATP | 2H | ∅ | 0.19 | ↑ | -0.66 | ↑↑↑ | -1.87 | ∅ | -0.17 | ↑↑↑ | -2.49 | | ↑↑ | -0.96 |
| *IL1B* | LPS | 1H | ↓↓↓ | 4.98 | ∅ | -0.32 | ∅ | -0.02 | ∅ | -0.16 | ∅ | 0.21 | | ∅ | 0.16 |
|  | R848 | 1H | ↓↓↓ | 5.00 | ∅ | -0.31 | ∅ | 0.02 | ∅ | 0.01 | ∅ | 0.28 | | ∅ | 0.28 |
|  | LPS/ATP | 1H | ↓↓↓ | 4.27 | ∅ | -0.32 | ∅ | 0.07 | ∅ | -0.23 | ∅ | 0.04 | | ∅ | -0.23 |
|  | LPS | 2H | ↓↓↓ | 4.72 | ∅ | 0.05 | ↓↓↓ | 0.83 | ∅ | -0.14 | ↓↓↓ | 1.12 | | ↓ | 0.41 |
|  | R848 | 2H | ↓↓↓ | 5.04 | ∅ | -0.40 | ∅ | 0.29 | ∅ | 0.05 | ∅ | 0.62 | | ∅ | 0.22 |
|  | LPS/ATP | 2H | ↓↓↓ | 3.76 | ∅ | -0.12 | ↓↓ | 0.74 | ∅ | -0.02 | ↓↓ | 0.83 | | ∅ | 0.16 |
| *IL6* | LPS | 1H | ↓↓↓ | 8.68 | ∅ | -0.35 | ∅ | 0.14 | ∅ | 0.27 | ∅ | 0.24 | | ∅ | 0.21 |
|  | R848 | 1H | ↓↓↓ | 8.21 | ∅ | -0.51 | ∅ | 0.19 | ∅ | 0.32 | ∅ | 0.28 | | ∅ | 0.16 |
|  | LPS/ATP | 1H | ↓↓↓ | 7.55 | ∅ | -0.74 | ∅ | 0.23 | ∅ | -0.02 | ∅ | -0.18 | | ∅ | -0.40 |
|  | LPS | 2H | ↓↓↓ | 7.31 | ∅ | 0.88 | ↓↓↓ | 1.76 | ∅ | 0.61 | ↓↓↓ | 2.80 | | ↓ | 1.18 |
|  | R848 | 2H | ↓↓↓ | 7.90 | ∅ | -0.03 | ∅ | 1.23 | ∅ | 0.83 | ↓↓ | 1.82 | | ∅ | 0.76 |
|  | LPS/ATP | 2H | ↓↓↓ | 5.91 | ∅ | 0.28 | ↓ | 1.13 | ∅ | 0.26 | ↓↓ | 1.30 | | ∅ | 0.44 |
| *NFKB1* | LPS | 1H | ∅ | 0.31 | ↑↑ | -0.52 | ∅ | 0.06 | ∅ | 0.06 | ∅ | -0.26 | | ∅ | -0.16 |
|  | R848 | 1H | ↓↓↓ | 0.91 | ↑↑ | -0.56 | ∅ | 0.16 | ∅ | 0.14 | ∅ | -0.07 | | ∅ | -0.08 |
|  | LPS/ATP | 1H | ∅ | -0.24 | ↑↑ | -0.55 | ∅ | 0.11 | ∅ | -0.04 | ∅ | -0.29 | | ∅ | -0.28 |
|  | LPS | 2H | ↓↓↓ | 2.09 | ∅ | -0.31 | ∅ | -0.06 | ∅ | -0.15 | ∅ | -0.15 | | ∅ | -0.12 |
|  | R848 | 2H | ↓↓↓ | 2.20 | ↑ | -0.44 | ∅ | -0.04 | ∅ | 0.18 | ∅ | -0.03 | | ∅ | 0.07 |
|  | LPS/ATP | 2H | ↓↓↓ | 1.65 | ∅ | -0.31 | ∅ | 0.07 | ∅ | 0.10 | ∅ | -0.14 | | ∅ | -0.05 |
| *NFKBIA* | LPS | 1H | ↓↓↓ | 3.03 | ∅ | -0.19 | ∅ | -0.19 | ∅ | -0.10 | ∅ | -0.11 | | ∅ | -0.06 |
|  | R848 | 1H | ↓↓↓ | 3.29 | ∅ | -0.12 | ∅ | -0.08 | ∅ | 0.03 | ∅ | 0.04 | | ∅ | 0.07 |
|  | LPS/ATP | 1H | ↓↓↓ | 2.79 | ∅ | -0.11 | ∅ | -0.15 | ∅ | -0.18 | ∅ | -0.15 | | ∅ | -0.22 |
|  | LPS | 2H | ↓↓↓ | 2.50 | ∅ | -0.02 | ∅ | 0.07 | ∅ | -0.13 | ∅ | 0.06 | | ∅ | -0.08 |
|  | R848 | 2H | ↓↓↓ | 2.52 | ∅ | -0.18 | ∅ | -0.06 | ∅ | 0.03 | ∅ | 0.01 | | ∅ | -0.04 |
|  | LPS/ATP | 2H | ↓↓↓ | 2.42 | ∅ | 0.01 | ∅ | 0.09 | ∅ | -0.00 | ∅ | 0.06 | | ∅ | -0.06 |
| *RELA* | LPS | 1H | ↑↑ | -0.46 | ↑ | -0.35 | ∅ | -0.07 | ∅ | 0.08 | ∅ | -0.13 | | ∅ | -0.02 |
|  | R848 | 1H | ∅ | 0.02 | ∅ | -0.32 | ∅ | 0.06 | ∅ | 0.07 | ∅ | 0.06 | | ∅ | -0.03 |
|  | LPS/ATP | 1H | ↑↑↑ | -0.84 | ↑ | -0.35 | ∅ | -0.07 | ∅ | 0.04 | ∅ | -0.13 | | ∅ | -0.18 |
|  | LPS | 2H | ↓↓↓ | 0.54 | ∅ | -0.00 | ↓ | 0.29 | ∅ | 0.01 | ∅ | 0.25 | | ∅ | 0.13 |
|  | R848 | 2H | ↓↓↓ | 0.68 | ↑ | -0.39 | ∅ | -0.07 | ∅ | -0.03 | ∅ | 0.01 | | ∅ | -0.17 |
|  | LPS/ATP | 2H | ↓ | 0.34 | ∅ | -0.25 | ∅ | 0.20 | ∅ | 0.16 | ∅ | 0.06 | | ∅ | 0.00 |
| *TLR4* | LPS | 1H | ↑↑ | -0.46 | ↑↑↑ | -0.70 | ↑↑ | -0.37 | ∅ | -0.02 | ↑↑ | -0.37 | | ∅ | -0.14 |
|  | R848 | 1H | ∅ | -0.18 | ∅ | -0.63 | ∅ | -0.55 | ∅ | -0.04 | ∅ | -0.32 | | ∅ | -0.21 |
|  | LPS/ATP | 1H | ↑↑↑ | -0.83 | ↑↑↑ | -0.75 | ↑↑ | -0.57 | ∅ | -0.10 | ↑↑ | -0.53 | | ∅ | -0.20 |
|  | LPS | 2H | ↓↓ | 0.42 | ↑↑↑ | -0.64 | ∅ | -0.11 | ∅ | 0.09 | ↑↑ | -0.37 | | ↑ | -0.27 |
|  | R848 | 2H | ↓ | 0.76 | ∅ | -0.46 | ∅ | 0.22 | ∅ | 0.40 | ∅ | 0.12 | | ∅ | 0.15 |
|  | LPS/ATP | 2H | ∅ | 0.28 | ↑↑ | -0.47 | ∅ | -0.21 | ∅ | -0.10 | ↑↑↑ | -0.69 | | ↑↑↑ | -0.59 |
| *TLR7* | LPS | 1H | ∅ | -0.27 | ∅ | 0.24 | ∅ | 0.07 | ∅ | 0.01 | ↓↓ | 1.23 | | ↓ | 0.98 |
|  | R848 | 1H | ∅ | 0.46 | ∅ | 0.21 | ∅ | -0.04 | ∅ | 0.04 | ↓↓↓ | 1.20 | | ↓↓ | 0.83 |
|  | LPS/ATP | 1H | ↑↑↑ | -1.34 | ∅ | 0.40 | ∅ | 0.26 | ∅ | -0.08 | ↓↓↓ | 1.18 | | ∅ | 0.31 |
|  | LPS | 2H | ∅ | 0.68 | ↓ | 1.14 | ↓ | 1.15 | ∅ | 0.63 | ↓↓↓ | 2.00 | | ↓↓ | 1.41 |
|  | R848 | 2H | ∅ | 0.06 | ∅ | 0.49 | ∅ | 0.31 | ∅ | 0.12 | ↓↓ | 0.77 | | ∅ | 0.41 |
|  | LPS/ATP | 2H | ∅ | -0.40 | ↓ | 0.63 | ∅ | 0.51 | ∅ | 0.36 | ↓↓↓ | 1.41 | | ↓↓ | 0.83 |
| *TLR8* | LPS | 1H | ↑↑ | -1.43 | ∅ | -0.26 | ∅ | -0.33 | ∅ | 0.11 | ∅ | -0.04 | | ∅ | 0.24 |
|  | R848 | 1H | ∅ | -0.60 | ∅ | -0.13 | ∅ | -0.40 | ∅ | -0.14 | ∅ | 0.02 | | ∅ | -0.01 |
|  | LPS/ATP | 1H | ↑↑↑ | -1.68 | ∅ | -0.24 | ∅ | -0.49 | ∅ | -0.04 | ∅ | -0.11 | | ∅ | 0.03 |
|  | LPS | 2H | ↑↑↑ | -1.76 | ∅ | 0.72 | ∅ | -0.26 | ∅ | 0.78 | ∅ | 0.46 | | ∅ | 0.96 |
|  | R848 | 2H | ↑ | -1.01 | ∅ | 0.41 | ∅ | 0.14 | ∅ | 0.02 | ↓ | 0.91 | | ∅ | 0.03 |
|  | LPS/ATP | 2H | ↑↑↑ | -2.91 | ∅ | 0.09 | ∅ | -0.52 | ∅ | -0.07 | ∅ | -0.22 | | ∅ | 0.14 |
| *TNF* | LPS | 1H | ↓↓↓ | 4.30 | ↓↓ | 0.61 | ∅ | 0.28 | ∅ | -0.08 | ↓↓↓ | 1.52 | | ↓↓↓ | 1.23 |
|  | R848 | 1H | ↓↓↓ | 4.85 | ∅ | 0.19 | ∅ | 0.16 | ∅ | 0.01 | ↓↓↓ | 1.08 | | ↓↓ | 0.94 |
|  | LPS/ATP | 1H | ↓↓↓ | 3.09 | ↓↓↓ | 0.86 | ∅ | 0.40 | ∅ | -0.29 | ↓↓↓ | 1.51 | | ↓↓ | 0.57 |
|  | LPS | 2H | ↓↓↓ | 3.40 | ↓↓↓ | 1.71 | ↓↓↓ | 1.07 | ∅ | -0.09 | ↓↓↓ | 2.26 | | ↓↓↓ | 1.67 |
|  | R848 | 2H | ↓↓↓ | 4.98 | ↓ | 0.63 | ↓↓↓ | 1.09 | ↓ | 0.67 | ↓↓↓ | 2.16 | | ↓↓↓ | 1.68 |
|  | LPS/ATP | 2H | ↓↓↓ | 2.51 | ↓↓↓ | 1.54 | ↓↓ | 0.69 | ∅ | 0.04 | ↓↓↓ | 1.89 | | ↓↓↓ | 1.40 |
| *IRF3* | R848 | 1H | ↑ | -0.83 | ∅ | -0.10 | ∅ | -0.05 | ∅ | -0.22 | ∅ | -0.61 | | ∅ | -0.55 |
|  |  | 2H | ∅ | -0.04 | ∅ | -0.10 | ∅ | 0.17 | ∅ | -0.07 | ∅ | 0.18 | | ∅ | -0.30 |
| *IRF7* | R848 | 1H | ∅ | -0.46 | ∅ | -0.00 | ∅ | 0.26 | ∅ | 0.11 | ∅ | -0.23 | | ∅ | -0.29 |
|  |  | 2H | ∅ | 0.66 | ∅ | -0.05 | ∅ | 0.18 | ∅ | 0.07 | ∅ | 0.26 | | ∅ | -0.03 |

Cord and adult blood (n=5 each, analyzed combined) was pretreated for 2 hours with PTX (200 μM), DEX (10^-7^ M), AZI (20 μM) or vehicle control (CTL), alone or in combination. Samples were stimulated with 10 ng/ml LPS, 1 µg/ml R848, or LPS followed by 5 mM ATP for inflammasome induction, and cultured for 1 hour or 2 hours at 37°C in 5% CO_2_. Mean ΔΔC_T_ values of samples undergoing different treatment conditions compared to TLRA stimulated samples (reference samples). ↑ significant upregulation, ↓ significant downregulation, and ∅ unchanged mRNA expression compared to reference samples, with the number of symbols representing the level of significance (p≤0.05, p≤0.01, and p≤0.001, respectively).

^a^ Some data were adapted from [20], *Speer EM, et al. Pediatr Res. 2017;81: 806-816*.

^b^ p-values were based on linear mixed model t-tests.
