# Supplementary material for: Pentoxifylline, dexamethasone and azithromycin demonstrate distinct age-dependent and synergistic inhibition of TLR- and inflammasome-mediated cytokine production in human newborn and adult blood in vitro
Source: PLoS One. 2018 May 1;13(5):e0196352. doi: 10.1371/journal.pone.0196352 (PMC5929513; doi:10.1371/journal.pone.0196352)
Supplement: S6 Table — (DOCX) [file pone.0196352.s013.docx]

S6 Table. Effects of combination treatment vs PTX alone on TLR- and/or inflammasome-induced mRNA expression in newborn and adult blood.

| **Gene** | **TLR agonist** | **mRNA expression changes of combination treatment vs PTX alone (Mean ΔΔCT)^*^** | | | | | | | |
| --- | --- | --- | --- | --- | --- | --- | --- | --- | --- |
|  |  | **1 hour stimulation** | | | | **2 hours stimulation** | | | |
|  |  | **PTX+DEX** | | **PTX+AZi** | | **PTX+DEX** | | **PTX+AZi** | |
| *CASP1* | LPS | ↓ | -0.38 | ↓↓ | -0.46 | ∅ | -0.14 | ∅ | -0.23 |
|  | R848 | ↓ | -0.41 | ∅ | -0.24 | ∅ | -0.25 | ∅ | -0.10 |
|  | LPS/ATP | ∅ | -0.16 | ∅ | -0.26 | ∅ | -0.07 | ∅ | -0.25 |
| *DUSP1* | LPS | ↑↑ | 0.55 | ∅ | 0.28 | ↑↑ | 0.54 | ∅ | 0.01 |
|  | R848 | ↑ | 0.46 | ∅ | 0.21 | ↑↑ | 0.66 | ∅ | -0.10 |
|  | LPS/ATP | ↑↑↑ | 0.40 | ∅ | 0.02 | ∅ | 0.14 | ∅ | -0.07 |
| *IL10* | LPS | ↑↑ | 1.25 | ∅ | 0.34 | ↑↑↑ | 1.54 | ∅ | -0.21 |
|  | R848 | ↑↑↑ | 1.46 | ∅ | 0.43 | ↑↑↑ | 1.82 | ∅ | 0.36 |
|  | LPS/ATP | ↑↑↑ | 1.04 | ∅ | -0.27 | ↑↑↑ | 1.83 | ∅ | 0.30 |
| *IL1B* | LPS | ↓↓↓ | -0.53 | ↓↓↓ | -0.48 | ↓↓↓ | -1.07 | ∅ | -0.36 |
|  | R848 | ↓↓↓ | -0.59 | ↓↓↓ | -0.59 | ↓↓↓ | -1.02 | ↓↓↓ | -0.62 |
|  | LPS/ATP | ↓↓↓ | -0.36 | ∅ | -0.09 | ↓↓↓ | -0.95 | ∅ | -0.28 |
| *IL6* | LPS | ↓↓ | -0.59 | ↓↓ | -0.55 | ↓↓↓ | -1.92 | ∅ | -0.30 |
|  | R848 | ↓↓ | -0.79 | ↓↓ | -0.67 | ↓↓↓ | -1.85 | ∅ | -0.79 |
|  | LPS/ATP | ↓↓ | -0.57 | ∅ | -0.35 | ↓↓↓ | -1.01 | ∅ | -0.15 |
| *NFKB1* | LPS | ∅ | -0.26 | ↓ | -0.37 | ∅ | -0.16 | ↓ | -0.19 |
|  | R848 | ↓ | -0.49 | ↓ | -0.48 | ↓ | -0.42 | ↓↓ | -0.52 |
|  | LPS/ATP | ∅ | -0.27 | ∅ | -0.27 | ∅ | -0.17 | ↓ | -0.26 |
| *NFKBIA* | LPS | ∅ | -0.07 | ∅ | -0.13 | ∅ | -0.08 | ∅ | 0.06 |
|  | R848 | ∅ | -0.16 | ∅ | -0.20 | ∅ | -0.19 | ∅ | -0.14 |
|  | LPS/ATP | ∅ | 0.04 | ∅ | 0.11 | ∅ | -0.06 | ∅ | 0.06 |
| *RELA* | LPS | ∅ | -0.22 | ↓ | -0.32 | ↓ | -0.25 | ∅ | -0.13 |
|  | R848 | ∅ | -0.38 | ∅ | -0.29 | ↓ | -0.40 | ∅ | -0.22 |
|  | LPS/ATP | ↓ | -0.23 | ∅ | -0.18 | ↓ | -0.31 | ∅ | -0.25 |
|  | | | | | | | | | |
| *TLR4* | LPS | ↓ | -0.34 | ↓↓↓ | -0.56 | ∅ | -0.27 | ↓ | -0.37 |
|  | R848 | ↓ | -0.31 | ↓↓ | -0.42 | ∅ | -0.58 | ↓ | -0.61 |
|  | LPS/ATP | ↓ | -0.22 | ↓↓↓ | -0.54 | ∅ | 0.22 | ∅ | 0.12 |
| *TLR7* | LPS | ↓↓↓ | -0.98 | ↓↓ | -0.73 | ↓↓ | -0.87 | ∅ | -0.27 |
|  | R848 | ↓↓↓ | -0.99 | ↓ | -0.62 | ∅ | -0.28 | ∅ | 0.08 |
|  | LPS/ATP | ↓↓↓ | -0.78 | ∅ | 0.09 | ↓↓ | -0.83 | ∅ | -0.20 |
| *TLR8* | LPS | ∅ | -0.21 | ↓↓↓ | -0.50 | ∅ | 0.26 | ∅ | -0.24 |
|  | R848 | ∅ | -0.15 | ∅ | -0.13 | ∅ | -0.50 | ∅ | 0.38 |
|  | LPS/ATP | ∅ | -0.13 | ↓↓ | -0.27 | ∅ | 0.31 | ∅ | -0.05 |
| *TNF* | LPS | ↓↓ | -0.91 | ↓ | -0.62 | ↓↓ | -0.55 | ∅ | 0.04 |
|  | R848 | ↓↓↓ | -0.89 | ↓↓ | -0.75 | ↓↓↓ | -1.53 | ↓↓↓ | -1.05 |
|  | LPS/ATP | ↓↓↓ | -0.65 | ∅ | 0.29 | ↓ | -0.35 | ∅ | 0.14 |
| *IRF3* | R848 | ∅ | 0.51 | ∅ | 0.44 | ∅ | -0.28 | ∅ | 0.20 |
| *IRF7* | R848 | ∅ | 0.22 | ∅ | 0.29 | ∅ | -0.31 | ∅ | -0.01 |

Cord and adult blood (n=5 each, analyzed combined) was pretreated for 2 hours with PTX (200 μM), DEX (10^-7^ M), or AZI (20 μM), alone or in combination. Samples were stimulated with 10 ng/ml LPS, 1 µg/ml R848, or LPS followed by 5 mM ATP for inflammasome induction, and cultured for 1 hour or 2 hours at 37°C in 5% CO_2_. Mean ΔΔC_T_ values of samples subjected to combination treatment compared to samples treated with PTX alone (reference samples). A linear mixed model was performed to analyze each gene, stimulation and time point independently. The covariance structure between treatment conditions within the same subject was modeled as compound symmetry. ↑ significant upregulation, ↓ significant downregulation, and ∅ unchanged mRNA expression compared to reference samples, with the number of symbols representing the level of significance (p≤0.05, p≤0.01, and p≤0.001, respectively).

^*^p-values were based on linear mixed model t-tests.
